# Golexanolone reduces glial activation in the striatum and improves non-motor and some motor alterations in a rat model of Parkinson's disease

**DOI:** 10.3389/fnagi.2024.1417938

**Published:** 2024-06-21

**Authors:** Paula Izquierdo-Altarejos, Yaiza M. Arenas, Mar Martínez-García, Lola Vázquez, Gergana Mincheva, Magnus Doverskog, Thomas P. Blackburn, Nicolaas I. Bohnen, Marta Llansola, Vicente Felipo

**Affiliations:** ^1^Laboratory of Neurobiology, Centro de Investigación Príncipe Felipe, Valencia, Spain; ^2^Umecrine Cognition AB, Solna, Stockholm, Sweden; ^3^Department of Radiology, University of Michigan, Ann Arbor, MI, United States; ^4^Neurology Service and GRECC, Veterans Administration Ann Arbor Healthcare System, Ann Arbor, MI, United States; ^5^Morris K. Udall Center of Excellence for Parkinson's Disease Research, University of Michigan, Ann Arbor, MI, United States; ^6^Parkinson's Foundation Center of Excellence, University of Michigan, Ann Arbor, MI, United States

**Keywords:** glial activation, GABAergic neurotransmission, motor impairment, cognitive impairment, 6-OHDA model, Parkinson's disease

## Abstract

**Background:**

Parkinson's disease (PD) affects more than 6 million people worldwide. Along with motor impairments, patients and animal models exhibiting PD symptoms also experience cognitive impairment, fatigue, anxiety, and depression. Currently, there are no drugs available for PD that alter the progression of the disease. A body of evidence suggests that increased GABA levels contribute to the reduced expression of tyrosine hydroxylase (TH) and accompanying behavioral deficits. TH expression may be restored by blocking GABA_A_ receptors. We hypothesized that golexanolone (GR3027), a well-tolerated GABA_A_ receptor-modulating steroid antagonist (GAMSA), may improve Parkinson's symptoms in a rat model of PD.

**Objectives:**

The aims of this study were to assess whether golexanolone can ameliorate motor and non-motor symptoms in a rat model of PD and to identify some underlying mechanisms.

**Methods:**

We used the unilateral 6-OHDA rat model of PD. The golexanolone treatment started 4 weeks after surgery. Motor symptoms were assessed using Motorater and CatWalk tests. We also analyzed fatigue (using a treadmill test), anhedonia (via the sucrose preference test), anxiety (with an open field test), and short-term memory (using a Y maze). Glial activation and key proteins involved in PD pathogenesis were analyzed using immunohistochemistry and Western blot.

**Results:**

Rats with PD showed motor incoordination and impaired locomotor gait, increased fatigue, anxiety, depression, and impaired short-term memory. Golexanolone treatment led to improvements in motor incoordination, certain aspects of locomotor gait, fatigue, anxiety, depression, and short-term memory. Notably, golexanolone reduced the activation of microglia and astrocytes, mitigated TH loss at 5 weeks after surgery, and prevented the increase of α-synuclein levels at 10 weeks.

**Conclusions:**

Golexanolone may be useful in improving both motor and non-motor symptoms that adversely affect the quality of life in PD patients, such as anxiety, depression, fatigue, motor coordination, locomotor gait, and certain cognitive alterations.

## 1 Introduction

Parkinson's disease (PD) affects over 6 million people worldwide and is expected to affect more in the upcoming decades (Dorsey et al., 2018). Patients with PD experience impaired motor coordination and locomotor gait, as well as other symptoms such as mild cognitive impairment, fatigue, anxiety, and depression, which strongly impair their quality of life (Lindgren and Dunnett, 2012; Kostić et al., 2016; Leão et al., 2021; Schneider et al., 2021; Tian et al., 2022; Zhang et al., 2023).

Currently, there are no drugs available that alter the progression of PD. Current symptomatic treatments, such as L-DOPA, provide limited relief and have side effects. L-DOPA (levodopa), a precursor of dopamine, is considered the most effective therapeutic option for patients with PD (Balestrino and Schapira, 2020): L-DOPA alleviates the main motor symptoms of the disease. However, up to 80% of patients develop uncontrollable abnormal involuntary movements (AIMs), known as L-DOPA-induced dyskinesia (LID). In addition, as the disease progresses, patients often encounter levodopa-refractory challenges such as postural instability, gait difficulties, such as falls or freezing of gait, and cognitive decline (Tran et al., 2018). Therefore, novel treatments to improve these levodopa-refractory motor and non-motor symptoms of PD are warranted.

The hallmark of any neurodegenerative disease is selective neuronal loss, which is associated with the activation of microglia and astrocytes (Dickson, 2012). Neuroinflammation, with the activation of microglia and astrocytes, plays a key role in the etiology of PD and other α-synucleinopathies (Kam et al., 2020; Stefanova, 2022; Chen et al., 2023). The motor symptoms of PD are generally attributed to nigrostriatal dopamine depletion, caused by extensive loss of dopaminergic neurons in the substantia nigra, resulting in the loss of tyrosine hydroxylase (TH), the main dopamine-producing enzyme (Zhou et al., 2022). However, the loss of TH may also be due to other causes and, under certain conditions, may be reversible. Several reports support the idea that enhanced GABAergic neurotransmission contributes to the pathogenesis of PD (PD) and associated motor symptoms (Lemos et al., 2016; Heo et al., 2020; Muñoz et al., 2020). Heo et al. (2020) showed that in animal models of PD, the levels of GABA are increased in activated astrocytes within the substantia nigra pars compacta. This increase is associated with the induction of an aberrant tonic GABA current in dopaminergic neurons, which is blocked by bicuculline, indicating enhanced activation of GABA_A_ receptors by tonically released astrocytic GABA. These authors also showed the presence of TH^−^/DCC^+^ neurons expressing dopamine decarboxylase (DCC) but not TH. They named these neurons “dormant neurons,” in which TH is not expressed due to the blockade of TH expression by the enhanced activation of GABA_A_ receptors. Heo et al. (2020) also showed that reducing GABA levels by inhibiting monoamine oxidase B (MAO_B_) with safinamide, or by reducing the activation of GABA_A_ receptor signaling through pharmacological blockade of Gabra5, restores TH loss and alleviates parkinsonian motor symptoms, and proposed that these treatments led to the recovery of TH expression in previously inactive neurons by reducing GABAergic neurotransmission.

Golexanolone is a well-tolerated novel drug in clinical development for neurological dysfunction in neurological and non-neurological diseases. Golexanolone reduces the activation of GABA_A_ receptors by acting on the neurosteroid binding site. The neurosteroids allopregnanolone and THDOC act as positive allosteric modulators of GABA_A_ receptors. Golexanolone is a GABA_A_ receptor-modulating steroid antagonist (GAMSA) (Johansson et al., 2016), which antagonizes the effects of allopregnanolone and THDOC on GABA_A_ receptors (Bengtsson et al., 2023), thus reducing the potentiation of GABA_A_ receptors in animal models and humans (Johansson et al., 2015, 2018; Arenas et al., 2023).

Golexanolone reduces glial activation and neuroinflammation and improves cognitive and motor function in rats with hyperammonemia and hepatic encephalopathy. It is considered a promising therapeutic drug for ameliorating neurological impairments in patients with hepatic encephalopathy and primary biliary cholangitis (PBC) (Johansson et al., 2015; Montagnese et al., 2021; Mincheva et al., 2022; Arenas et al., 2023).

Most non-motor symptoms in PD patients are related to α-synuclein pathology (Jellinger, 2011). Cui et al. (2023) reported that the presence of 6-OHDA increases α-synuclein content in the lesioned striatum. It has been proposed that there is an interplay between glial activation, neuroinflammation, and α-synuclein (Balzano et al., 2023). Consequently, we analyzed the effects of golexanolone treatment on α-synuclein accumulation.

We hypothesized that golexanolone might enhance TH expression in dormant neurons, thereby improving certain motor and non-motor deficits in a rat model of PD. We used the 6-OHDA rat model, as described by Carvalho et al. (2013). The aims of this study were to assess: (1) whether daily treatment with golexanolone improves motor symptoms (motor coordination and locomotor gait) and non-motor symptoms (cognitive function, fatigue, anxiety, and depression) in the 6-OHDA rat model of PD and (2) whether these improvements are associated with reductions in microglia and astrocyte activation, enhancements in TH expression in the striatum, and decreases in alpha-synuclein content.

## 2 Materials and methods

### 2.1 The rat model of PD: unilateral 6-OHDA lesion

Eight-week-old Wistar-Han male rats (Charles River, Barcelona) were housed, two per cage, under standard laboratory conditions: a 12-h light-dark cycle, 22°C room temperature, 55% relative humidity, and *ad libitum* access to food and water. The experimental protocols were approved by the Comite de Etica y Experimentación Animal (CEEA) of our center and by the Conselleria de Agricultura, Generalitat Valenciana. All procedures were performed according to the Directive of the European Commission (2010/63/EU) on the care and management of experimental animals and complied with the ARRIVE guidelines for animal research. The unilateral 6-OHDA lesion procedure, as described by Carvalho et al. (2013), was conducted under isoflurane anesthesia (5% for induction, followed by a maintenance dose of 2%).

The rats were secured in a stereotaxic frame with non-traumatic ear bars (Stoelting, USA) and unilaterally injected (the right hemisphere) with either vehicle (the sham group) or 6-OHDA hydrochloride (Sigma, USA) (6-OHDA group) directly into the medial forebrain bundle (the coordinates used relative to Bregma were AP = −4,4 mm; ML = −1,0 mm; DV = −7,8 mm, following the guidelines by Paxinos and Watson). At a rate of 1 μl/min, the rats in the sham group received 2 μl of 0.2 mg/ml ascorbic acid dissolved in 0.9% NaCl. Conversely, the 6-OHDA rats were injected with 2 μl 6-OHDA hydrochloride (4 μg/μl) combined with 0.2 mg/ml ascorbic acid in 0.9% NaCl. After the injection, the syringe was left in place for an additional 10 min to allow diffusion of the substance.

### 2.2 Apomorphine turning behavior test

To validate the model, the apomorphine-induced turning behavior was evaluated 3 weeks after surgery, following the protocols as established by Torres and Dunnett (2011). For this test, the rats were subcutaneously injected in the neck with 0.1 mg/kg of apomorphine hydrochloride (Sigma-Aldrich) dissolved in 1% ascorbic acid in 0.9% NaCl. Shortly after the injection, the rats were placed in individual bows. Five min after the injection, their rotations were recorded and tracked using Anymaze software for 15 min. The net number of rotations was calculated by subtracting the number of ipsilateral rotations from the number of contralateral rotations. Only the rats displaying more than four net rotations per minute were included in the study for further analysis.

### 2.3 Experimental groups

**Figure 1:**
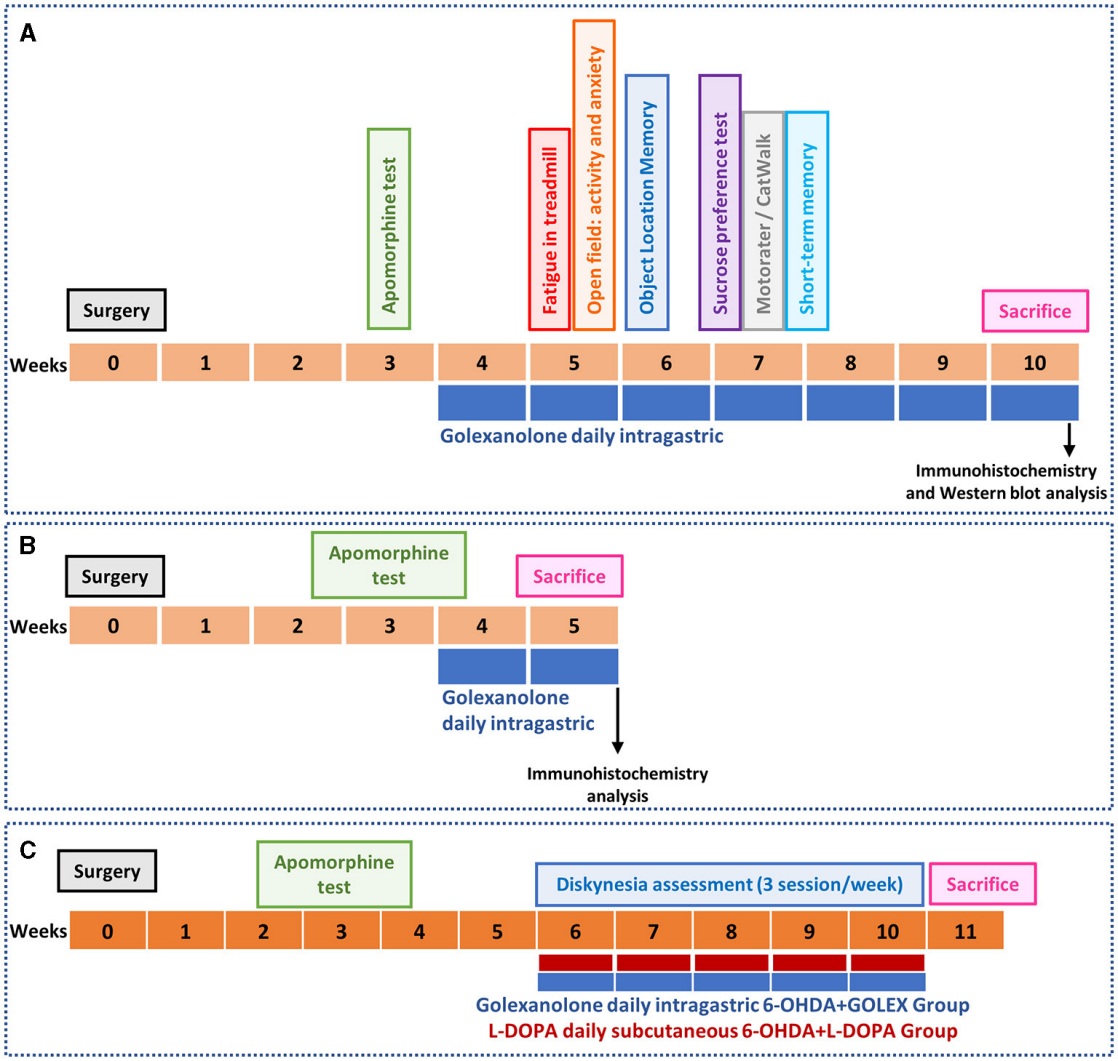
Experimental design. The unilateral injection of 6-OHDA was used a model of Parkinson's diseases. The apomorphine-induced rotation test was performed 3 weeks after surgery to select successfully operated rats. **(A)** In one set of rats (*n* = 8–10 per group), golexanolone treatment started at week 4 after surgery and different motor and non-motor parameters were evaluated: fatigue (treadmill), anxiety and ambulatory activity (open field test), object location memory, anhedonia (sucrose preference test), motor coordination (Motorater), locomotor gait (CatWalk) and short-term memory. Rats were sacrificed 10 weeks after surgery to extract the brains for immunohistochemistry and western blot analysis. **(B)** A different set of rats (*n* = 6 per group) didn't perform the behavioral tests and was sacrificed at 5 weeks after surgery to evaluate glial activation and TH parameters by immunohistochemistry at a shorter time. **(C)** A third set of rats (*n* = 12 per group) started golexanolone and DOPA treatment at weeks 6 after surgery and the induction of dyskinesia was evaluated during 5 weeks.

The experimental design is summarized in Figure 1. The rats were divided into the following experimental groups: (1) Sham-operated rats receiving CAPMUL as a vehicle (SHAM VH), (2) Sham-operated rats treated with golexanolone (SHAM GR), (3) 6-OHDA rats receiving CAPMUL as vehicles (PARK VH); and (4) 6-OHDA rats treated with golexanolone (PARK GR). Two sets of experiments were conducted with these groups. One set of rats (8–10 rats per group) performed the behavioral tests described below and was subsequently euthanized 10 weeks after surgery (Figure 1A). The second set of rats (six per group) was euthanized 5 weeks after surgery for the purpose of conducting immunohistochemistry analysis (detailed in Figure 1B). This bifurcated approach allowed for the assessment of both the short-term and long-term effects of the treatments within the experimental framework.

In an additional experiment, the induction of dyskinesia by golexanolone and L-DOPA was analyzed (Figure 1C). This experiment included the following groups: (1) 6-OHDA rats treated with golexanolone and subcutaneously injected with saline and (2) 6-OHDA rats treated with L-DOPA and receiving CAPMUL (*n* = 12 per group). Treatments started at week 6 after surgery, and dyskinesias were evaluated over the course of the following 5 weeks.

### 2.4 Treatments

Golexanolone [40 mg/ml in CAPMUL (Capmul^®^ MCM, Glycerol Monocaprylocaprate (Type I) from ABITEC Corporation (Janesville, WI, USA)], or CAPMUL as a vehicle, was administered daily using intragastric probes at a dose of 50 mg/kg (1.25 ml/kg), following the methodology of Johansson et al. (2015). Additionally, L-DOPA methyl ester-HCl (Sigma) was administered daily via subcutaneous injection at a dosage of 6 mg/kg, combined with the DOPA-decarboxylase inhibitor benserazide-HCl at 10 mg/ml (Sigma), according to the protocols described by Lundblad et al. (2002). For all the behavioral tests described below, golexanolone was administered 1 h before the commencement of the tests.

### 2.5 Fatigue assessment

Physical fatigue was evaluated using a treadmill, based on a modified procedure from Butterworth et al. (2009). The rats had a single day of pre-training to acclimate to the equipment and were tested the following day. The pre-training consisted of a 3-min exploration phase where the treadmill belt remained stationary and inclined at 0 degrees. Then, the treadmill was started at 10 cm/s for 5 min and then increased to 20 cm/s for another 5 min. On the test day, the rats were placed on the treadmill, now inclined at 5 degrees, with the speed gradually increasing up to 30 cm/s over a 5-min period. After reaching this speed, it was maintained for an additional 15 min. Throughout this test, the Anymaze video tracking software was used to record and quantify the time spent on the grid (shock zone). The rats that remained in the shock zone without showing an intention to walk before the end of the test were removed from the treadmill, and the total time spent on the treadmill was recorded.

### 2.6 Evaluation of anxiety in the open-field test

The open-field test is used to evaluate general locomotor activity and anxiety levels in rodents. Rodents display a natural dislike for brightly lit open areas. Decreased anxiety levels lead to increased exploratory behavior, more locomotion, and a preference to stay in the center of the field, whereas stressed or anxious rats show a preference to stay in peripheral zones with more darkness. The open-field test was conducted according to the methodology described by Ujvári et al. (2022). The open-field arena consists of a cube (100 × 100 × 50 cm), and it was divided into a central zone and an outer zone in the periphery. Rat activity was recorded with the Anymaze video tracking for 5 min after the animal was placed in the center of the cube. The software measured and analyzed the total time spent in the center zone (s) and total traveled distance (cm) over the course of 5 min.

### 2.7 Anhedonia: sucrose preference test

To assess the presence of anhedonic behavior, a characteristic behavior in depression, the rats underwent the sucrose preference test, as described by Tadaiesky et al. (2008). Each rat was provided with two water bottles on the extreme sides of the cage during the 24-h training phase to allow the rats to adapt to drinking from two bottles. After the training, one bottle of the bottles was randomly replaced with a bottle containing a 1% sucrose solution, and 24 h later, the bottles were reversed to avoid perseveration effects. The use of a 48-h testing period allowed us to preclude any effects of neophobia, artifactual bias toward any particular side, and perseveration effects. After 20 h of water deprivation, the animals were presented with pre-weighed bottles, one containing tap water and the other filled with 1% sucrose solution (in the same type of water). Two h later, sucrose and water intake were measured to determine the difference between the initial and final weight of the bottle. Sucrose preference was calculated according to the formula: sucrose preference = [sucrose intake/(sucrose intake + water intake)] × 100.

### 2.8 Motor function

Motor coordination and locomotor gait parameters were assessed after 4 weeks of golexanolone administration.

#### 2.8.1 Motor coordination in the motorater

A kinematic analysis of motor coordination was conducted using the MotoRater apparatus (TSE Systems, Germany), as described by Zörner et al. (2010). The system consisted of a horizontal ladder that the animals had to cross (referred to as a “run”) to reach their own cage. During this process, the rats' movements were recorded from three different angles. Three uninterrupted runs were recorded for each rat. The runs were analyzed by counting and classifying the steps as correct or wrong paw placements (slips or misses). The results are expressed as a percentage of total steps and are the mean of the three runs.

#### 2.8.2 Footprint analysis of locomotor gait in the CatWalk™

CatWalk XT is a video-based system for automatic gait analysis (Noldus, Wageningen, the Netherlands). The system consisted of an enclosed walkway on a glass plate that the animals had to cross from side to side (each cross is considered a run) to reach a dark box that connected to their housing cage, where they felt secure. The glass had a green light internally reflected that could only escape and be detected in the areas where the animal was touching it. When a part of the animal was in contact with the glass, a digital image was created. The software allowed one to discriminate between paws (front or hind and left or right). Three runs were recorded each day for 2 days. The data were analyzed using the CatWalk^TM^ analysis software (v 7.1) and were the average of the six runs. The software analyzed the step cycle in the run, that is, the time between two consecutive initial contacts of the same paw, and that is divided into Stand + Swing. Stand is the duration of contact of a paw with the glass plate, and Swing (%) is the duration of no contact of a paw with the glass plate as a percentage of the step cycle. The regularity index represents the percentage of normal step sequence patterns relative to the total number of paw placements and can be used as a measure of inter-paw coordination, usually at 100% in healthy animals. Print positions are the distance between the position of the hind paw and that of the previously placed front paw on the same side of the body and in the same step cycle.

The stand index is a measure of the speed at which the paw loses contact with the glass plate during motion. “Max Intensity At (%)” refers to the point in time during the run, expressed as a percentage of the total stand duration, when the paw's contact with the surface reaches its peak intensity. Swing speed is the speed of the paw during a swing. Stride length is the distance between two successive placements of the same paw. The duty cycle (%) is another crucial metric; it represents the stand duration as a percentage of the entire step cycle (as opposed to the swing percentage), and the dual stance refers to the intervals within a step cycle when both hind paws are in contact with the ground simultaneously. This is further broken down into Initial Dual Stance, which is the first instance of simultaneous ground contact by both hind paws during the step cycle, and Terminal Dual Stance, which is the second occurrence of such contact within the same cycle. These metrics provide detailed insights into the gait dynamics and motor coordination of the subjects).

Short-term spatial recognition memory was analyzed using a Y-maze. The rats were placed into one arm (start arm) and allowed to explore the maze with one arm closed for 2 min (the training trial) two times, with 1 min of inter-trial interval. In the test trial, performed after 1 min, the rats were allowed to explore both arms for 2 min. The number of entries and the time spent in each arm were recorded, and a discrimination ratio [(time spent in the novel arm–time spent in the familiar arm)/total time spent in the two arms] was calculated.

#### 2.8.3 Analysis of dyskinesias

The appearance of abnormal involuntary movements (AIMs) was followed for 5 weeks (three times per week). The rats were placed individually in transparent plastic cages and recorded for 1 min every 20th min at 20–180 min after the injection of L-DOPA or vehicle, as done by Lundblad et al. (2002). As described before (Cenci and Lundblad, 2007; Mincheva et al., 2022), AIMs were classified into four subtypes: (1) axial, dystonic posturing or choreiform twisting of the neck and upper body toward the side contralateral to the lesion; (2) limb, abnormal, purposeless movements of the forelimb and digits contralateral to the lesion; (3) orolingual, empty jaw movements and contralateral tongue protrusion; and (4) locomotive, increased locomotion toward the contralateral side. Each of these four subtypes was scored on a severity scale from 0 to 3 (0 = not present during the observation time, 1 = present during less than half of the observation time, 2 = present during more than half of the observation time, and 3 = present all the time). The theoretical maximum score that could be accumulated by one animal in one testing session for each AIM subtype was 27 (maximum score per observation point = 3 × number of observation points = 9).

### 2.9 Immunohistochemistry

For immunohistochemistry studies, the rats were anesthetized with sodium pentobarbital and transcardially perfused with 4% paraformaldehyde in 0.1 M phosphate buffer (pH = 7.4). The brains were extracted and post-fixed in the same fixative solution for 24 h. The tissue was embedded in paraffin, and 5 μm sections were cut and mounted on coated slides. The samples from the rats analyzed 5 weeks after surgery were cut longitudinally, while those from the rats after 10 weeks were cut transversally. Three sections from different parts of the striatum, covering the whole medium-lateral (for longitudinal sections) or whole anteroposterior (for transversal sections) striatum, were taken. The sections were sequentially incubated with 3% H_2_O_2_, blocking serum, and the following primary antibodies at 4°C overnight: Iba1 (1:300, 019–19741 Wako); GFAP (1:300, G3893 Sigma), tyrosine hydroxylase (1:500, ab112 Abcam), or α-synuclein (1:100, 10842-1-AP Proteintech). Then, slides were incubated with biotinylated secondary antibodies (1:200, Vector Laboratories), goat anti-mouse, or goat anti-rabbit for 1 h at room temperature. The signal was amplified using the Vectastain ABC kit (Vector Laboratories). The chromogenic reaction was performed using a DAB kit (Abcam), and finally, the slides were counterstained with Mayer's hematoxylin (DAKO). The sections were scanned with an Aperio Versa system (Leica Biosystems, Germany) for further image analysis.

### 2.10 Analysis of microglia and astrocyte activation

For this analysis, fields at 40 × magnification were acquired for the scanned slides using the software ImageScope64. From each rat, 8–10 images were taken from the three sections in the striatum region (either contralateral or ipsilateral). Microglia activation was analyzed by measuring the perimeter of individual Iba1-stained cells using Image-Pro Plus (v.6.0) software. We used the measurement of the microglia perimeter as a measure of the acquisition of an amoeboid shape by the microglia when their phagocytic activity increases, which is indicative of reactive microglia. In this state, the microglia's ramifications are shortened, leading to a decrease in the total perimeter. This method, and perimeter decrease in general, has been extensively used to quantify microglia activation and coincide with an increased number of microglia cells (Gonzalez-Perez et al., 2002; Rodrigo et al., 2010; Agusti et al., 2017; Ullah et al., 2020). Astrocyte activation was assessed by measuring the total GFAP-stained area in the three sections per rat using ImageJ software. For both microglia and astrocytes, the results were the average of all images analyzed from each rat, which were expressed as a percentage of shams.

### 2.11 Analysis of tyrosine hydroxylase and α-synuclein content

For this analysis, fields containing the striatum region (either contralateral or ipsilateral) were captured from the scanned slides at lower magnification for each rat. The optical density of TH, or α-synuclein, was quantified using ImageJ software. The entire striatal area was manually selected using the ROI (Region of Interest) manager function in ImageJ. The inverted values of mean intensity were recorded, and the results represent the average values derived from the three different slides for each rat and were expressed as a percentage of the values obtained from sham-operated control rats.

### 2.12 Analysis of protein content by Western blot

The animals were euthanized by decapitation 10 weeks after surgery. The striata from both the contralateral and ipsilateral were dissected, homogenized in five volumes of lysis buffer (50 mM TRIS–HCl pH 7.5, 50 mM NaCl, 10 mM EGTA, 5 mM EDTA, with protease and phosphatase inhibitors) using sonication, and then centrifuged at 13,000 × g for 10 min. The total protein content in the supernatant was determined using the bicinchoninic acid method (BCA, from PIERCE). Samples (30 μg) from both hemispheres (injected and contralateral) in the same gel were subjected to electrophoresis and immunoblotting, following the procedure outlined by Felipo et al. (1988). The membranes were probed with the following primary antibodies: tyrosine hydroxylase (1:4,000, ab112 Abcam), α-synuclein (1:1,000, 10842-1-AP Proteintech), and β-actin (1:5,000, ab6276 Abcam) as loading control. The membranes were then scanned using a ScanJet 5,300C (Hewlett-Packard, Amsterdam, the Netherlands), and band intensities were quantified using Alpha Imager 2,200 version 3.1.3 (Alpha Innotech Corporation). Band intensities were normalized to the β-actin intensity on the same membrane to ensure accurate quantification. The results were expressed as a percentage of those obtained from sham-operated controls, facilitating comparative analysis.

### 2.13 Statistical analysis

Data are expressed as mean ± SEM. All statistical analyses were conducted using GraphPad Prism software v. 9.0. Data were tested for normality (Kolmogorov–Smirnov or Shapiro-Wilk test) and for homogeneity of variances. Statistical analysis was carried out using a two-way ANOVA, which considered two factors: the first factor being sham vs. 6-OHDA lesion, and the second factor being vehicle vs. Golexanolone treatment. *Post-hoc* comparisons between groups were conducted using Tukey's or Fisher's multiple comparison tests, depending on the specific requirements of the data. Statistics data are indicated in each figure legend.

## 3 Results

### 3.1 The 6-OHDA rats show increased fatigue, anxiety-like behavior, and anhedonia. Golexanolone improves all these alterations but not the decreased ambulatory activity

**Figure 2:**
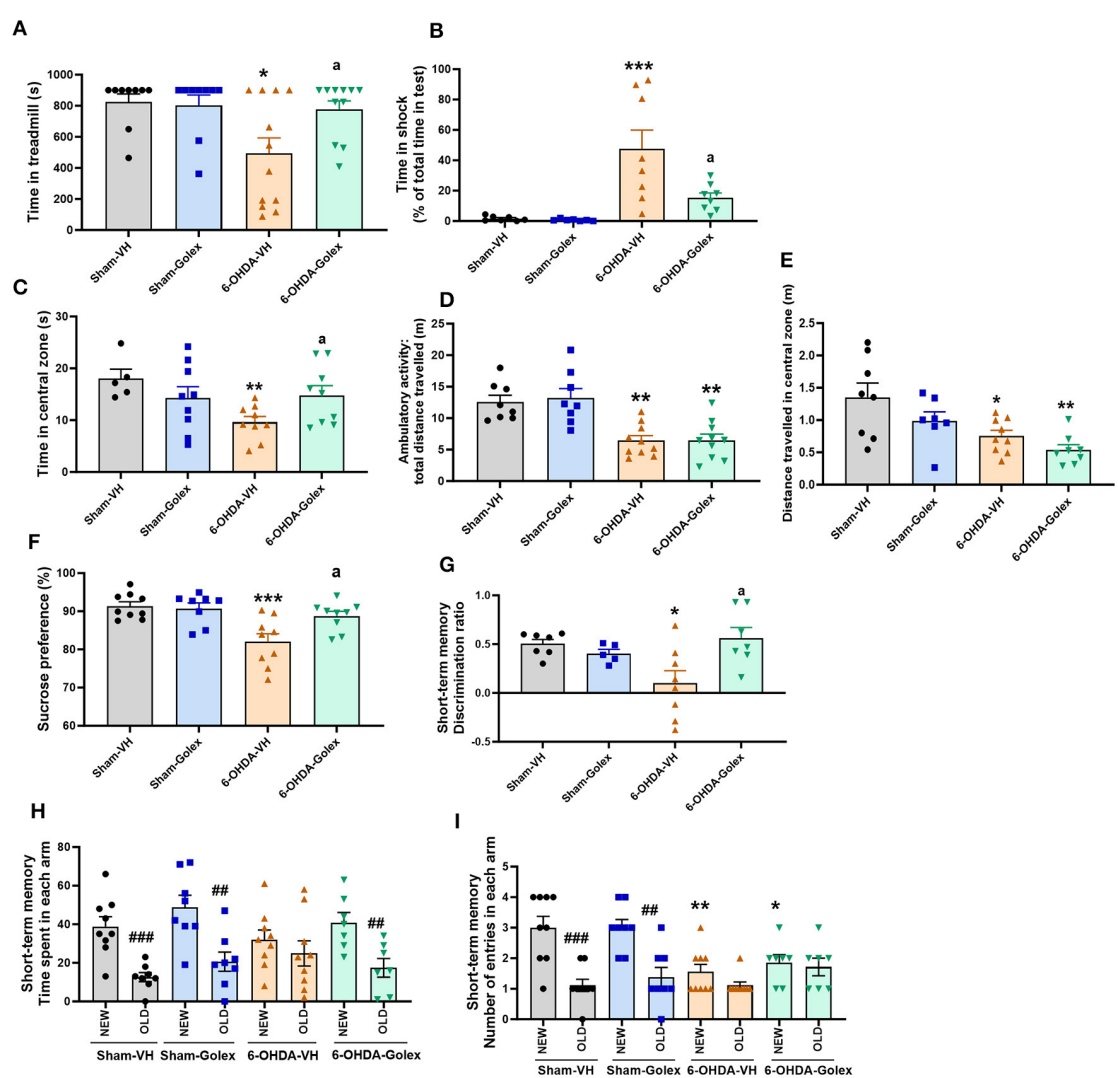
Golexanolone treatment reverses fatigue, anxiety, anhedonia and short-term memory deficits in 6-OHDA rats. Fatigue was evaluated in the treadmill: **(A)** time in treadmill [6-OHDA effect was significant (*p* = 0.024) whereas treatment effect (*p* = 0.094) and interaction were not statistically significant; Tukey's test was used for multiple comparisons] **(B)** time in shock zone, expressed as percentage of total time of the test (effect of 6-OHDA (*p* = 0.0002), of golexanolone treatment (*p* = 0.024), and interaction (*p* = 0.032) were all statistically significant; Tukey's test was used for multiple comparisons). Anxiety was evaluated in the open field during 5 min: time spent in the central zone of the box [effect of 6-OHDA injection was significant (*p* = 0.045), as well as interaction (*p* = 0.027), whereas treatment effect was not statistically significant; Fisher's LSD test was used for multiple comparisons] (**C)**, ambulatory activity, total distance traveled, was also evaluated in the open field (only effect of 6-OHDA injection was significant (*p* < 0.0001); Tukey's test was used for multiple comparisons) **(D)** and distance in the central zone [effect of 6-OHDA injection was significant (*p* = 0.001), but treatment effect does not reach significance (*p* = 0.051) and interaction was not significant; Tukey's test was used for multiple comparisons] **(E)**. Anhedonia, evaluated by the sucrose preference test, was expressed as percentage of sucrose intake [effect of 6-OHDA was statistically significant (*p* = 0.0009) as well as interaction effect (*p* = 0.023), whereas treatment effect not reached significance (*p* = 0.058); Tukey's test was used for multiple comparisons] **(F)**. Short-term memory evaluated in a Y-maze was measured with a discrimination ratio [injection of 6-OHDA do not induce significant effect but there is a significant interaction (*p* = 0.011) and treatment effect not reach significance (*p* = 0.085); Tukey's test was used for multiple comparisons] **(G)**. In **(H)** is shown the time spent in each arm. In all groups except in the 6-OHDA group without golexanolone treatment, time spent in the new arm is significantly higher than in the old arm (one-way ANOVA followed by Tukey's test was used). In **(I)** we also show number of entries in each arm, which was higher to the new arm in shams groups but not in 6-OHDA groups, in which total number of entries is significant lower due to lower activity (one-way ANOVA followed by Tukey's test was used). Data are the mean ± SEM of 7–12 rats per group. Values significantly different from SHAM-VH rats are indicated by asterisks (*p < 0.05, **p < 0.01, ***p < 0.001), values significantly different from 6-OHDA-VH are indicated by a (a = *p* < 0.05) and values significant different between new and old arm in the Y-maze are indicated with # (^##^ *p* < 0.01; ^###^ *p* < 0.001) (VH, vehicle and Golex, golexanolone).

Fatigue was evaluated using a treadmill. When the rats became tired and stopped running, they received a mild electric shock. The 6-OHDA rats spent less time on the treadmill than the sham rats (494 ± 100s vs. 824 ± 53 s, *p* < 0.05) (Figure 2A) and endured more time receiving shocks (48 ± 12 s vs. 1.65 ± 0.62 s, *p* < 0.01) (Figure 2B), which reflects increased fatigue. Treatment with golexanolone notably ameliorated these effects in the 6-OHDA rats, as shown by an increase in the duration they remained active on the treadmill (775 ± 56 s) (Figure 2A) and a decrease in the time spent in the shock zone (15 ± 3 s, *p* < 0.05 compared to 6-OHDA rats without treatment) (Figure 2B).

Anxiety was evaluated in an open arena for 5 min by measuring the time that the rat remained in the central zone instead of close to the walls of the box, where the rats felt safer. The 6-OHDA rats remained for less time than sham rats in the central zone (9.6 ± 1.1 s vs. 18 ± 2 s, *p* < 0.01), indicating more anxiety-like behavior. Treatment with golexanolone completely reversed anxiety (15 ± 2 s, *p* < 0.05 compared with the 6-OHDA rats without treatment) (Figure 2C). Total distance traveled (a measure of ambulatory activity) was also reduced in the 6-OHDA-injected rats compared to the sham rats (6.4 ± 0.8 m vs. 13 ± 1 m, *p* < 0.01) and was not improved by golexanolone (6.5 ± 0.8 m vs. 13 ± 1 m, *p* < 0.01 compared with the sham rats) (Figure 2D). We also analyzed the distance traveled in the central zone, which is also reduced in 6-OHDA rats, both with and without golexanolone treatment (Figure 2E). This indicates that the 6-OHDA-treated rats moved less but spent more time in the center, which is the measure of anxiety-like behavior, and is reversed by golexanolone. The sucrose preference test evaluates anhedonia, a symptom of depression. As shown in Figure 2F, the 6-OHDA rats showed reduced preference for the sucrose solution compared with the sham rats (82 ± 2 % vs. 91 ± 1 %, *p* < 0.001), indicating anhedonia (depression) in the rats, which was completely reversed by golexanolone (89 ± 1 %, *p* < 0.05 compared with the 6-OHDA rats without treatment) (Figure 2F).

### 3.2 The 6-OHDA rats show reduced short-term memory, which is improved by golexanolone

Short-term memory deficits were observed in the 6-OHDA rats, as evaluated using the Y maze. The discrimination ratio in these rats was significantly lower compared to the sham rats (0.1 ± 0.13 vs. 0.5 ± 0.05, *p* < 0.05) (Figure 2G), indicating impaired short-term memory. Golexanolone treatment effectively normalized this measure (0.6 ± 0.1, *p* < 0.05 compared with the untreated 6-OHDA rats) (Figure 2G). Figure 2H further reveals that the 6-OHDA rats spent a similar amount of time exploring both the new and old arms of the maze, unlike the other groups, which showed a significant preference for the newly opened arm. Additionally, the number of entries into each arm was significantly reduced in the 6-OHDA rats, regardless of whether they were treated with golexanolone, which reflects a decrease in overall activity that was not ameliorated by the treatment (Figure 2I).

### 3.3 6-OHDA rats show motor incoordination and impaired locomotor gait. Golexanolone improves motor incoordination and locomotor gait

**Figure 3:**
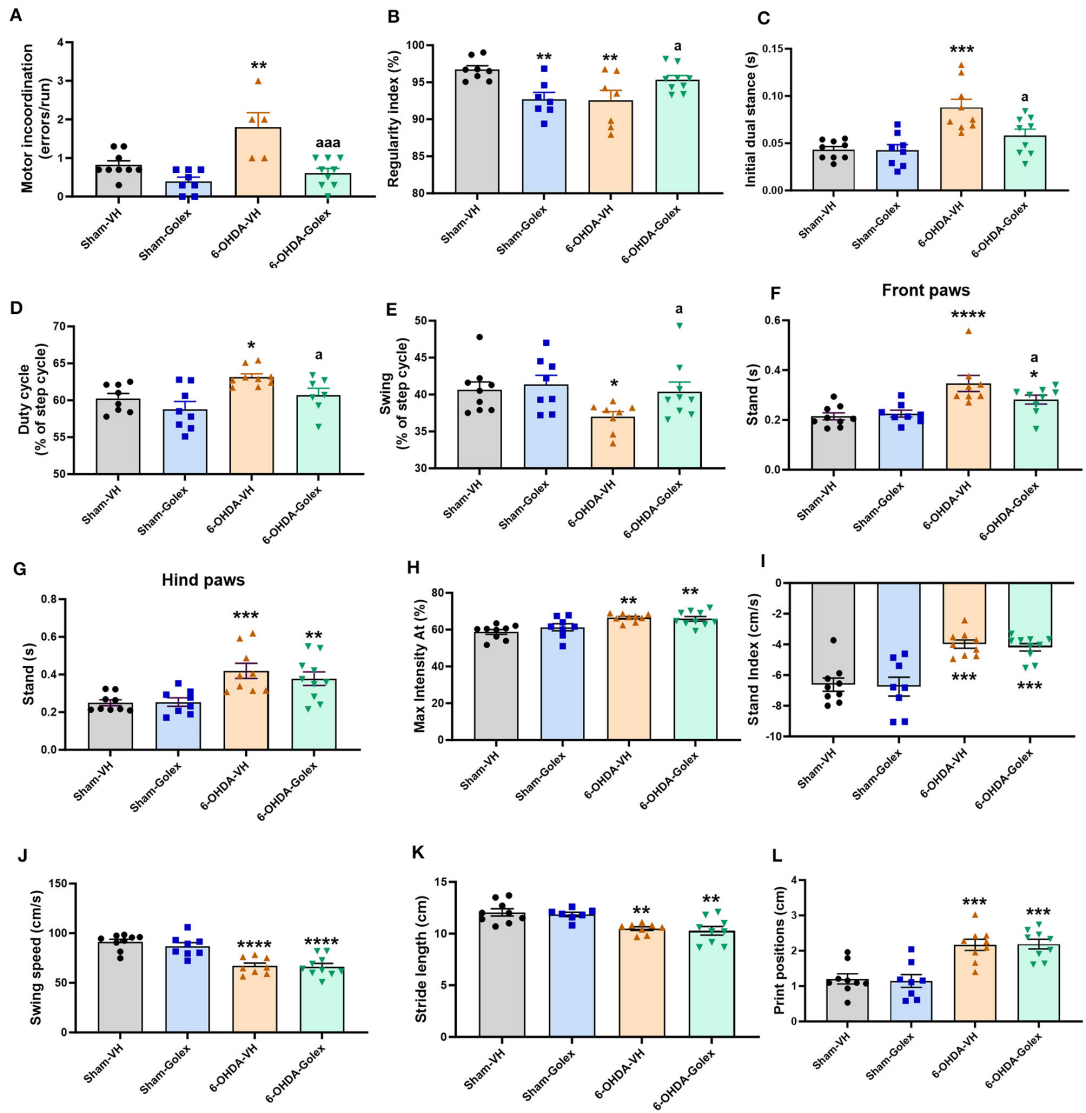
Golexanolone treatment reverses motor incoordination and some locomotor gait deficits in 6-OHDA rats. **(A)** Motor incoordination: average of errors/run for each rat in the Motorater test. Two-way ANOVA indicated significant effects of 6-OHDA (*p* = 0.0012), of golexanolone treatment (*p* = 0.0001) and also a significant interaction (*p* = 0.027); Tukey's test was used for multiple comparisons. Different parameters evaluating locomotor gait were measured in the CatWalk: **(B)** regularity index [the interaction was significant (0.0005), although 6-OHDA and golexanolone treatment did not induce significant effects; Fisher's LSD test was used for multiple comparisons], **(C)** print positions were increased in 6-OHDA groups [group effect was significant (*p* = 0.0001) but not treatment effect or interaction; Tukey's test was used for multiple comparisons], **(D)** stand index [only 6-OHDA effect was significant (*p* = 0.0001); Tukey's test was used for multiple comparisons], **(E)** stand is shown separated for front and hind paws, as the effects were different; for front paws there is a significant effect of 6-OHDA injection but not of treatment, but interaction was close of significance (*p* = 0.077); Fisher's LSD test was used for multiple comparisons, **(F)** for hind paws only group effect was significant (*p* < 0.0001); Fisher's LSD test was used for multiple comparisons, **(G)** Max Intensity At as percentage of stand is also increased in 6-OHDA injected rats [only significant effect of 6-OHDA injection was found (*p* = 0.0001); Tukey's test was used for multiple comparisons], **(H)** swing (as percentage of the step cycle), statistic data were: *p* = 0.05 for 6-OHDA effect, *p* = 0.079 for treatment effect and no significant interaction; Fisher's LSD test was used for multiple comparisons, **(I)** swing speed is clearly decreased in 6-OHDA injected rats and not affected by golexanolone [only 6-OHDA effect was significant (*p* < 0.0001); Tukey's test was used for multiple comparisons], **(J)** stride length is decreased in 6-OHDA injected groups (*p* < 0.0001 for 6-OHDA effect and treatment effect or interaction were no significant; Tukey's test was used for multiple comparisons) **(K)** duty cycle, as percentage of the step cycle [effect of 6-OHDA injection is significant (*p* = 0.0043) and also effect of golexanolone treatment (*p* = 0.018) but not significant interaction was found; Fisher's LSD test was used for multiple comparisons], **(L)** initial dual stance [significant effect of 6-OHDA (*p* < 0.0001) and of golexanolone treatment (*p* = 0.028) were found, as well as a significant interaction (*P* = 0.034); Tukey's test was used for multiple comparisons]. Data are the mean ± SEM of 8–10 rats per group. Values significantly different from SHAM-VH rats are indicated by asterisks (*p < 0.05, **p < 0.01, ***p < 0.001, ****p < 0.0001) and values significantly different from 6-OHDA-VH are indicated by a (a = *p* < 0.05, aaa = *p* < 0.001) (VH, vehicle and Golex, golexanolone).

The 6-OHDA rats showed motor incoordination in the motorater, with an increased number of errors (1.8 ± 0.8 vs. 0.8 ± 0.1, *p* < 0.01 compared with the sham rats). Golexanolone treatment completely restored motor coordination in the 6-OHDA rats, reducing the number of errors to 0.6 ± 0.1 (*p* < 0.001 compared with the 6-OHDA rats without treatment; Figure 3A). Another indicator of motor coordination is the regularity index analyzed in the catwalk. The 6-OHDA rats showed a reduced regularity index compared to the sham rats (92.5 ± 1.4 % vs. 97 ± 0.5 %, *p* < 0.01), indicating motor incoordination, which was improved by golexanolone treatment (95.3 ± 0.6, *p* < 0.05 compared with the 6-OHDA rats without treatment) (Figure 3B).

We also analyzed different parameters to evaluate locomotor gait on the catwalk. The 6-OHDA rats showed increased initial dual stance (duration of contact with the ground of the two hind or front paws at the same time in each step) compared with the sham rats (0.08 ± 0.017 s vs. 0.044 ± 0.011 s, *p* < 0.01) (Figure 3C) and duty cycle [duration of contact of the paw with the ground, expressed as percentage of Step Cycle (the opposed to Swing percentage)] in the front paws (62 ± 2 % vs. 58 ± 2 %, *p* < 0.05) (Figure 3D). 6-OHDA rats showed a reduced swing percentage (the time a paw is not in contact with the ground, expressed as a percentage of Step Cycle) compared with the sham rats (37 ± 2 % vs. 41 ±3 %, *p* < 0.05) (Figure 3E). Treatment with golexanolone reversed these changes in initial dual stance (0.062 ± 0.017s, *p* < 0.05), duty cycle (56 ± 4 %, *p* < 0.01), and swing percentage (40 ± 4 %, *p* < 0.05) in the 6-OHDA rats (Figures 3C–E). Stand (the duration of contact of a paw with the glass plate) is increased in the 6-OHDA rats, and it is reduced by golexanolone treatment in the front but not in the hind paws (Figures 3F, G). However, there are also some parameters of locomotor gait that are altered in the 6-OHDA rats and are not improved by treatment with golexanolone: max intensity at % (Figure 3H), stand index (Figure 3I), swing speed (Figure 3J), stride length (Figure 3K), and print positions (Figure 3L).

### 3.4 Golexanolone treatment does not induce dyskinesia in 6-OHDA rats, while L-DOPA treatment induces it

**Figure 4:**
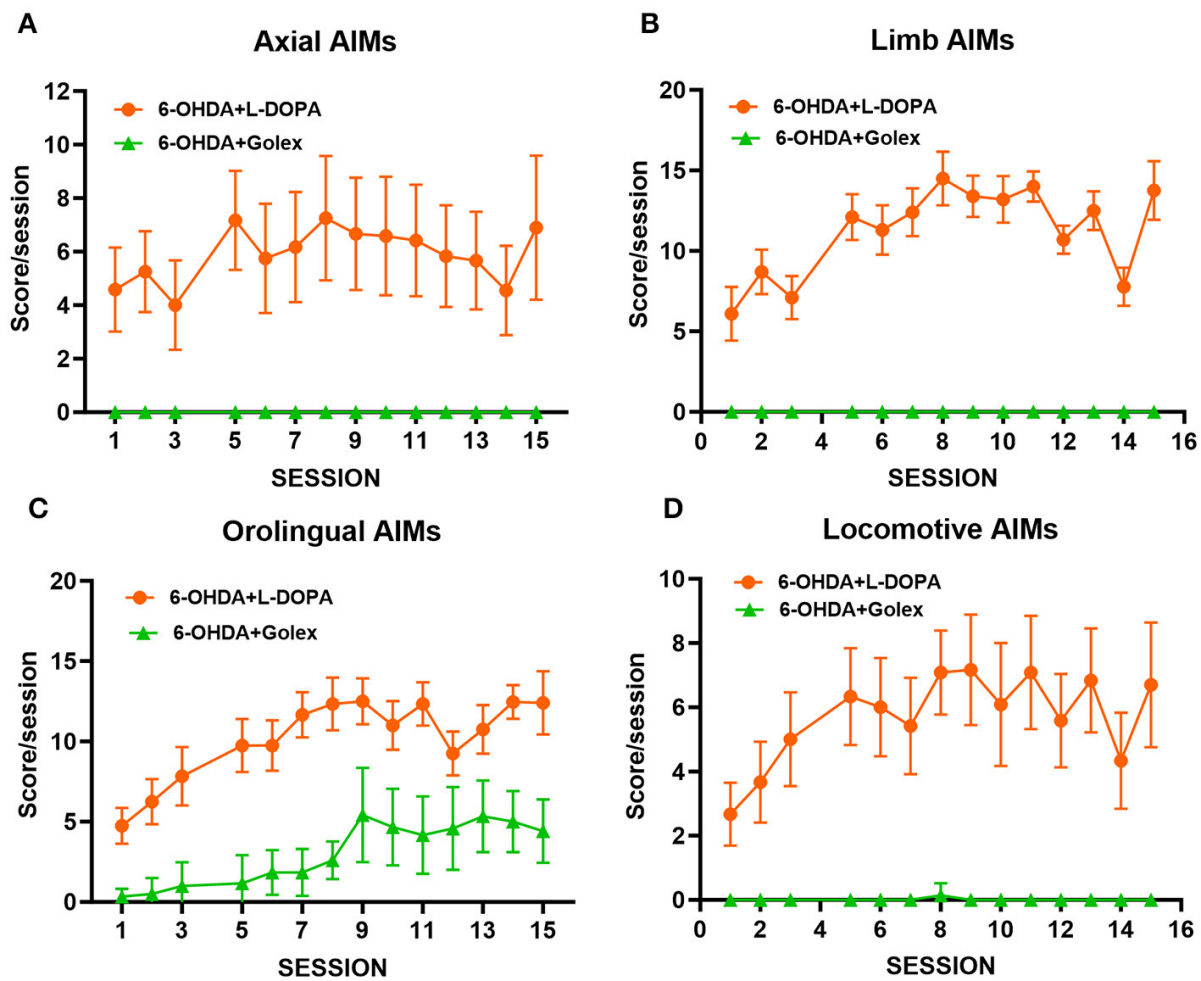
Golexanolone treatment only induces mild orolingual dyskinesias in 6-OHDA rats, unlike the general and strong dyskinesias induced by L-DOPA. Different types of dyskinesia were followed after golexanolone or L-DOPA treatment for 5 weeks, 3 sessions/week, for a total of 15 sessions. The score/session is showed for each of the four subtypes of AIMs evaluated: **(A)** axial, **(B)** limb, **(C)** orolingual, and **(D)** locomotive. Data are the mean ± SEM of 10–12 rats per group.

Different types of dyskinesia were monitored after administering golexanolone or L-DOPA treatment for 5 weeks at a frequency of 3 sessions/week, for a total of 15 sessions. L-DOPA treatment led to the onset of four subtypes of AIMs evaluated: axial, limb, orolingual, and locomotive (Figure 4). The intensity of AIMs increased during the initial sessions and then stabilized around sessions 7–8. In contrast, golexanolone treatment did not induce axial, limb, or locomotive AIMs (Figures 4A, B, D). There was a mild induction of orolingual AIMs in the 6-OHDA rats treated with golexanolone, but this was significantly less severe compared to the AIMs induced by L-DOPA (*p* < 0.0001) (Figure 4C).

### 3.5 Tyrosine hydroxylase content is decreased in the striatum of the 6-OHDA rats. Golexanolone treatment partially reduces this decrease at 5 weeks after surgery

Immunohistochemistry of TH was performed at 5 and 10 weeks after surgery and 2 and 6 weeks of treatment, respectively. TH immunostaining was quantified in both the right (injured) and left hemispheres.

**Figure 5:**
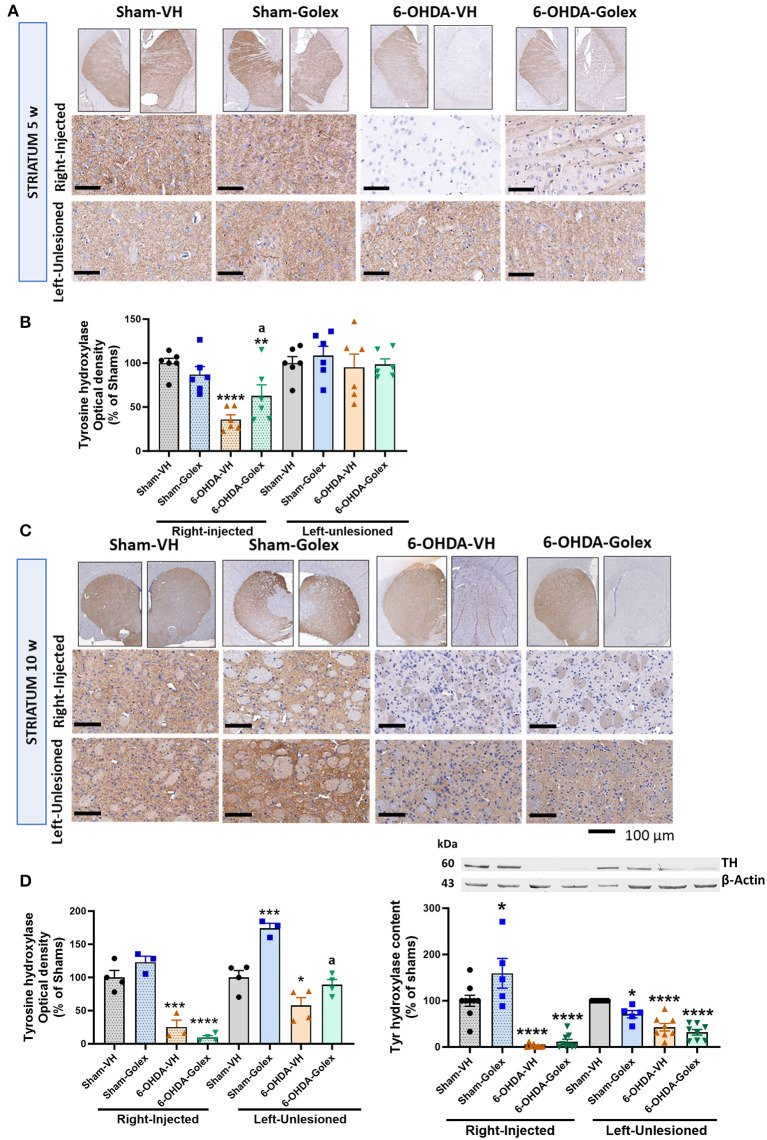
Tyrosine hydroxylase content is reduced in the striatum of 6-OHDA rats and golexanolone treatment partially restores it at 5 weeks after surgery. Representative images of immunohistochemistry staining against tyrosine hydroxylase (TH) in the ipsilateral **(right)** and contralateral **(left)** striatum at 5 **(A)** and 10 **(C)** weeks after surgery. Scale bar = 100 μm, as indicated in the figure. TH staining optical density in striatum, expressed as percentage of shams at 5 **(B)** and 10 **(D)** weeks after surgery. Data are the mean ± SEM of 6 rats per group in B and 3-4 rats per group in **(D)**. Data were analyzed by Two-way ANOVA, independently in the right and the left striatum: **(B)** in the injected striatum 6-OHDA effect was significant (*p* < 0.0001) and there is also significant interaction between 6-OHDA injection and treatment (*p* = 0.02) but treatment effect was not significant; in the contralateral striatum no significant differences were found; multiple comparisons were performed with Fisher's LSD test, **(D)** in the lesioned part 6-OHDA induced significant effect (*p* < 0.0001), but not golexanolone treatment, although interaction was almost significant (*p* = 0.053); Tukey's test was used for multiple comparisons; in the contralateral striatum both 6-OHDA effect (*p* < 0.0001) and treatment effect (*p* = 0.0003) were significant and interaction *p*-value was 0.055; multiple comparisons were performed with Fisher's LSD test. TH content was also analyzed by western blot **(E)**, in the injected hemisphere 6-OHDA effect (*p* < 0.0001) was significant as well as treatment effect (*p* = 0.014) and interaction *p*-value was *p* = 0.06; Tukey's test was used for multiple comparisons; in the contralateral hemisphere also 6-OHDA effect (*p* < 0.0001) and treatment effect (*p* = 0.0057) were significant but not the interaction; Tukey's test was used for multiple comparisons. Values significantly different from SHAM-VH rats are indicated by asterisks (*p < 0.05, **p < 0.01, ***p < 0.001, ****p < 0.0001) and values significantly different from 6-OHDA-VH are indicated by a (a *p* < 0.05).

At 5 weeks after surgery, TH staining was reduced in the right injured striatum of the 6-OHDA rats (36 ± 4 % of the sham rats, *p* < 0.0001), whereas the left striatum did not show any significant reduction (97 ± 13% of the sham rats) (Figures 5A, C). Golexanolone treatment significantly rescued the TH staining in the 6-OHDA rats (62 ± 11% of the sham rats, *p* < 0.05 compared with the 6-OHDA-untreated rats and *p* < 0.01 compared with the sham rats) (Figures 5A, B).

At 10 weeks after surgery, the 6-OHDA rats showed a larger reduction of TH staining in the injured striatum (25 ± 11% of shams, *p* < 0.001), and golexanolone treatment no longer prevented this TH loss (10 ± 3% of the sham rats, *p* < 0.0001) (Figures 5B, D). At this time, the left striatum also showed a significant reduction in TH in the 6-OHDA rats (58 ± 12% of shams, *p* < 0.05). This reduction in TH was prevented in the rats treated with golexanolone (89 ± 8 % of shams, *p* < 0.05 compared with the 6-OHDA-untreated rats) (Figures 5C, D). TH content was also analyzed by western blot in the striatum at 10 weeks after surgery. The results obtained were essentially equal to those obtained by immunohistochemistry except for the unlesioned hemisphere of the sham rats treated with golexanolone, likely due to variability between different rats. Both in the injected and contralateral hemispheres, TH was reduced in the 6-OHDA rats and was not affected by golexanolone treatment, as observed using immunohistochemistry (Figure 5E).

### 3.6 Alpha-synuclein is increased in the striatum of 6-OHDA rats at 10 weeks after surgery. Golexanolone treatment prevented this increase

**Figure 6:**
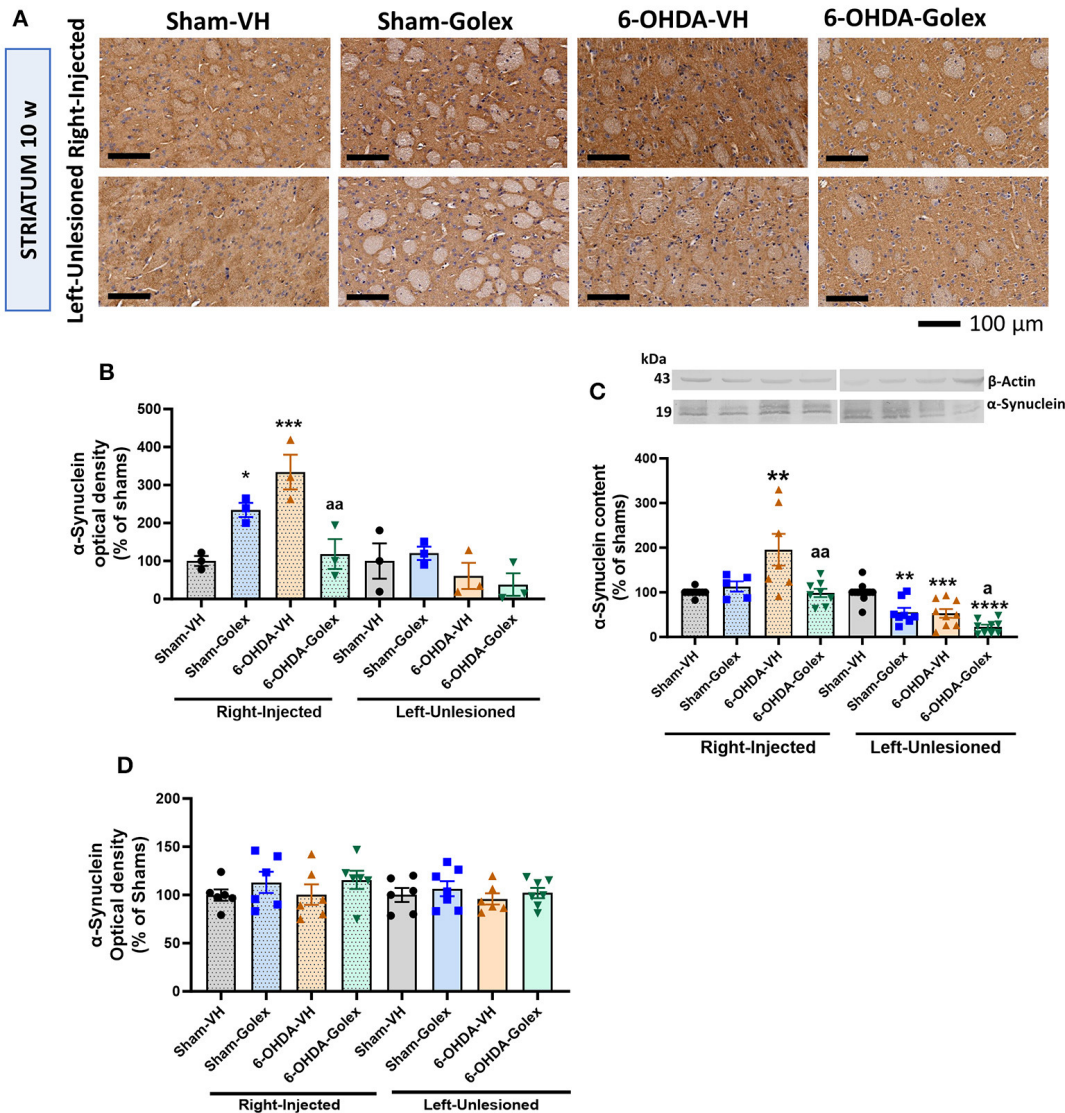
α-synuclein content is increased in the striatum of 6-OHDA rats and golexanolone treatment normalizes it at 10 weeks after surgery. **(A)** Representative images of immunohistochemistry staining against α-synuclein in the ipsilateral **(right)** and contralateral **(left)** striatum at 10 weeks after surgery. Scale bar = 100 μm, as indicated in the figure. Quantification of α-synuclein content (optical density in the immunohistochemistry) in 3–4 rats per group at ten **(B)** and five **(D)** weeks after surgery. Data are the mean ± SEM and were analyzed by Two-way ANOVA; at ten weeks, in the injected hemisphere not significant effect of 6-OHDA or treatment was found but interaction was significant (*p* = 0.0006); in the contralateral striatum no significant effects were found; multiple comparisons were performed with Fisher's LSD test; at 5 weeks, no significant difference was found between the groups. The content of α-synuclein at 10 weeks was also analyzed by Western blot in 8–9 rats per group **(C)** expressed as percentage of shams (mean ± SEM); in the injected striatum 6-OHDA effect (*p* = 0.035), treatment effect (*p* = 0.029) and interaction (*p* = 0.0057) were all significant; in the unlesioned striatum, also significant effects of 6-OHDA (*p* < 0.0001) and of treatment with golexanolone (*p* < 0.0001) were found but not significant interaction; Tukey's test was used for multiple comparisons. Values significantly different from SHAM-VH rats are indicated by asterisks (*p < 0.05, **p < 0.01, ***p < 0.001, ****p < 0.0001) and values significantly different from 6-OHDA-VH are indicated by a (a *p* < 0.05, aa *p* < 0.01).

The α-synuclein immunostaining was significantly increased in the injured striatum of the 6-OHDA rats at 10 weeks after surgery (334 ± 46% of shams, *p* < 0.001), and golexanolone treatment prevented this increase in the 6-OHDA rats (118 ± 39 % of shams, *p* < 0.01 compared with the 6-OHDA-untreated rats) (Figures 6A, B). The content of α-synuclein analyzed by immunohistochemistry in the striatum of the 6-OHDA rats was not altered in the untreated or treated rats 5 weeks after surgery (Figure 6D).

Similar results were obtained when the α-synuclein content was analyzed using Western blot. α-synuclein was also increased (195 ± 35% of shams, *p* < 0.05) in the injured striatum of the 6-OHDA rats at 10 weeks after surgery, and this increase was prevented by golexanolone (99 ± 9% of the sham rats, *p* < 0.05 compared with the 6-OHDA-untreated rats) (Figure 6C), as we observed using immunohistochemistry. In the contralateral striatum, a significant reduction of α-synuclein content was observed in the 6-OHDA rats but also in the sham rats and especially in the 6-OHDA rats treated with golexanolone (Figure 6C). This effect in the contralateral striatum can also be observed using immunohistochemistry, but it was not as evident and not statistically significant.

### 3.7 Microglia is activated in the striatum of 6-OHDA rats. Golexanolone treatment prevented this activation at 5 but not at 10 weeks after surgery

As an approximation to analyze the role of neuroinflammation in the beneficial effects of golexanolone in this Parkinson's model, we evaluated glial activation in the striatum.

**Figure 7:**
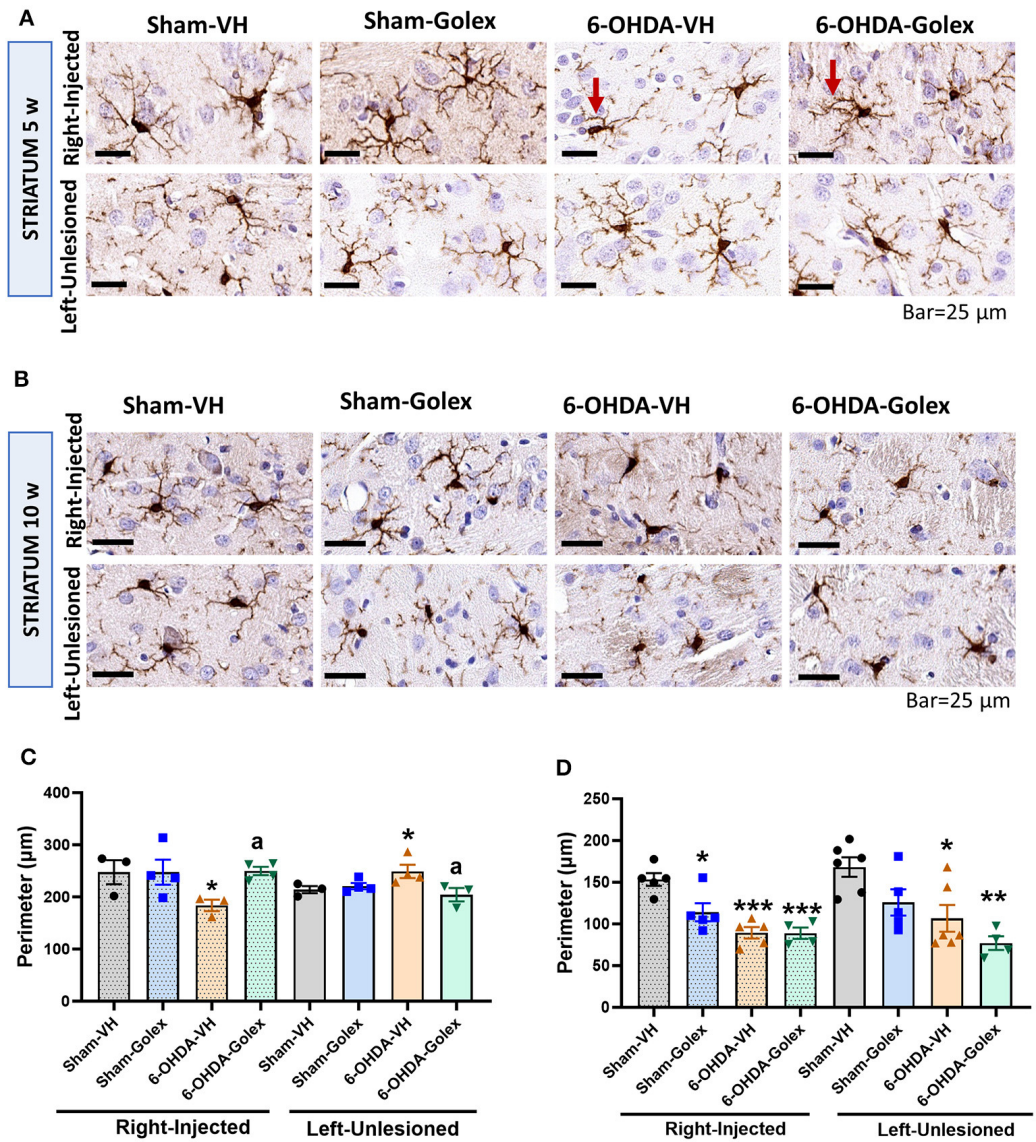
Microglia is activated in the striatum of 6-OHDA rats and golexanolone treatment normalizes it at 5 weeks after surgery. Representative images of immunohistochemistry staining against Iba1 in the ipsilateral **(right)** and contralateral **(left)** striatum at 5 **(A)** and 10 **(B)** weeks after surgery. Scale bar = 25 μm, as indicated in the figure. Arrows indicate examples of activated (ameboid) microglia cell in 6-OHDA group at 5 weeks and of a more branched (non-activated) microglial cell in the 6-OHDA group treated with golexanolone, indicating reversal of microglia activation by golexanolone. Perimeter of the Iba1 stained cells at 5 **(C)** and 10 **(D)** weeks after surgery. Data are the mean ± SEM of 3–6 rats per group and were analyzed by Two-way ANOVA. In **(C)**, injected hemisphere, no significant effects of 6-OHDA or treatment, or interaction, were found, but significant effects results from Fisher's LSD multiple comparisons test; in the contralateral hemisphere interaction was significant (*p* = 0.033) and multiple comparisons were also performed with Fisher's LSD test. In **(D)**, 6-OHDA injection has significant effect in the ipsilateral striatum (*p* < 0.0001), as well as treatment (*p* = 0.032) and interaction between group and treatment was also significant (*p* = 0.036). In the contralateral striatum the effect of 6-OHDA was also significant (*p* = 0.0012) and the treatment (*p* = 0.021), but not significant interaction exists; Tukey's test was used for multiple comparisons. Values significantly different from SHAM-VH rats are indicated by asterisks (*p < 0.05, **p < 0.01, ***p < 0.001) and values significantly different from 6-OHDA-VH are indicated by a (a *p* < 0.05).

Microglia activation is reflected in a decrease in the perimeter of microglia cells stained with Iba1 using immunohistochemistry. Microglia was significantly activated in the 6-OHDA rats in the injured striatum at 5 weeks (217 ± 8 μm compared with 254 ± 1 μm in the sham rats, *p* < 0.05) and at 10 weeks after surgery (87 ± 10 μm compared with 145 ± 8 μm in the sham rats, *p* < 0.05) (Figure 7). At 10 weeks, microglia was also activated in the contralateral striatum (84 ± 3 μm compared with 165 ± 18 μm in the sham rats, *p* < 0.05) (Figures 7B, D). Golexanolone significantly reduced microglia activation in the injured striatum at 5 weeks (250 ± 8 μm, *p* < 0.05 compared with the 6-OHDA-untreated rats) but not at 10 weeks in any hemisphere (Figure 7).

### 3.8 Astrocytes are activated in the striatum of the 6-OHDA rats 5 weeks after surgery. Golexanolone treatment prevented this activation

**Figure 8:**
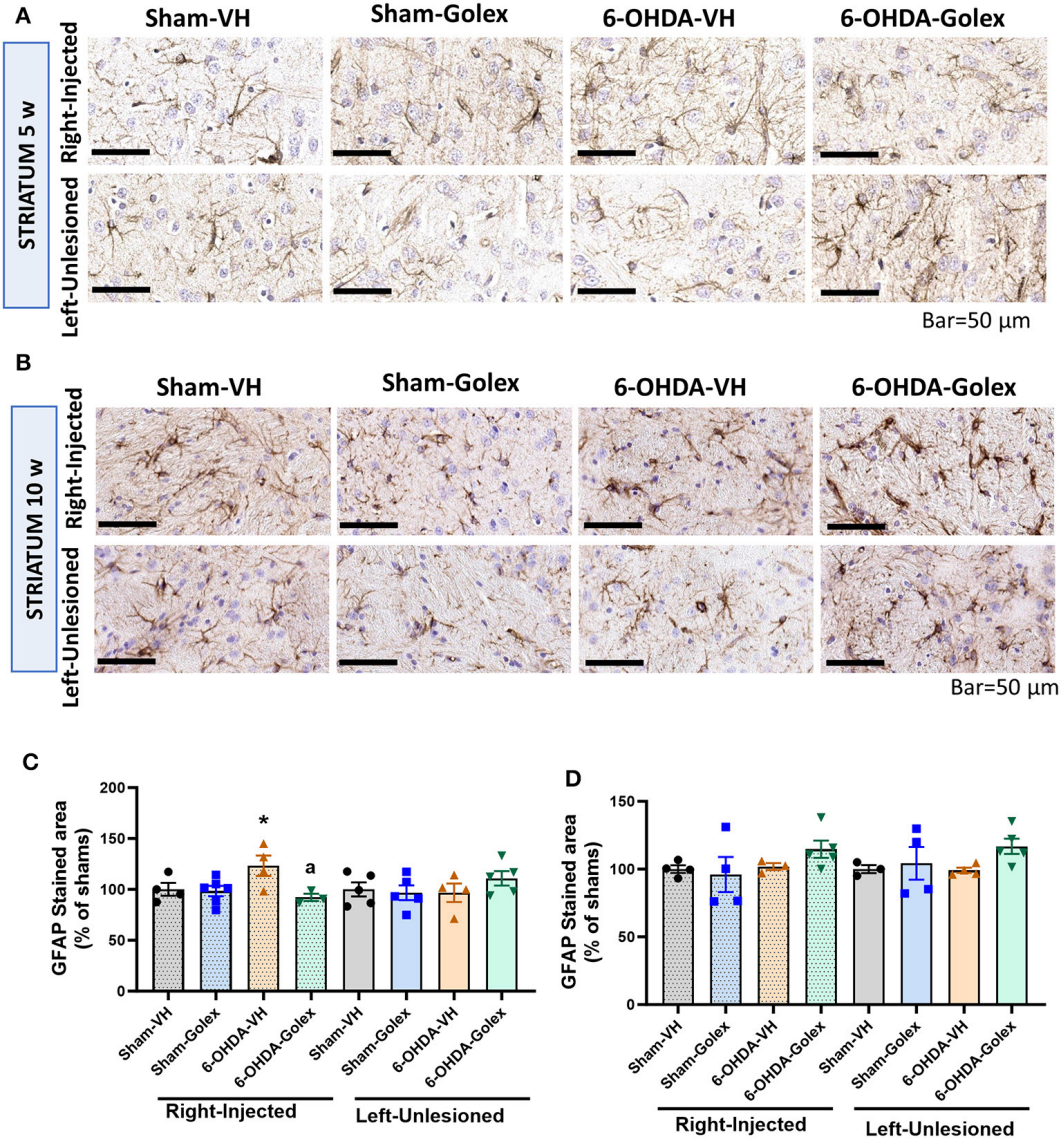
Astrocytes are activated in the striatum of 6-OHDA rats at 5 weeks after surgery and golexanolone treatment reduces this activation. Representative images of immunohistochemistry staining against GFAP in the ipsilateral **(right)** and contralateral **(left)** striatum at 5 **(A)** and 10 **(B)** weeks after surgery. Scale bar = 50 μm, as indicated in the figure. GFAP stained area in striatum, expressed as percentage of shams at **(C)** 5 and **(D)** 10 weeks after surgery. Data are the mean ± SEM of 3–5 rats per group and were analyzed by Two-way ANOVA; in C, in the injected striatum, significant effect of golexanolone treatment (*p* = 0.035) was found, but not of 6-OHDA injection, and interaction *p*-value was *p* = 0.054; in the contralateral striatum no significant differences were found; in **(D)** no significant differences were found in any hemisphere; multiple comparisons were performed with Fisher's LSD test. Values significantly different from SHAM-VH rats are indicated by asterisks (*p < 0.05) and values significantly different from 6-OHDA-VH are indicated by a (a *p* < 0.05).

Astrocyte activation in the striatum was analyzed using immunohistochemistry. An increased percentage of the area stained by GFAP reflects the activation of astrocytes. Astrocytes were significantly activated in the 6-OHDA rats in the injured striatum at 5 weeks (123 ± 10% of the sham rats, *p* < 0.05), and golexanolone reversed this increase (92 ± 4 % of the sham rats, *p* < 0.05 compared with the 6-OHDA-untreated rats) (Figures 8A, C). At 10 weeks, astrocyte activation was no longer present in the 6-OHDA rats (102 ± 3% of the sham rats) (Figures 8B, D). No differences were found in the contralateral striatum regarding astrocyte activation.

## 4 Discussion

The results reported here show that golexanolone has beneficial effects on both the motor and non-motor symptoms of PD. Golexanolone improves motor coordination and some parameters of locomotor gait, fatigue, anxiety, depression, and short-term memory deficits. Altogether, these beneficial effects would significantly improve the quality of life of patients with PD. It is also noteworthy that golexanolone improves all these varieties of symptoms without inducing any relevant dyskinesia, in contrast with L-DOPA. L-DOPA alleviates the main motor symptoms and is considered the most effective treatment for patients with PD. However, most patients develop dyskinesia, with uncontrollable abnormal involuntary movements that severely reduce their quality of life (Tran et al., 2018; Balestrino and Schapira, 2020).

Concerning motor symptoms, golexanolone improves motor coordination, fine motor kinematics, and gait performance, as reflected by the improved performance in the Motorater test and in some parameters of the Catwalk test such as regularity index, initial dual stance, percentage of swing, and duty cycle. Golexanolone does not seem to significantly improve bradykinesia, as reflected by the lack of improvement in ambulatory activity or in the step cycle, swing speed, or stride length in the catwalk.

Concerning freezing of gait (FoG), there is not a single locomotor gait parameter in rats that could be considered a direct measurement of FoG. It has been proposed that dual stance, as analyzed in the Catwalk, would reflect the FoG (Xiao et al., 2017; Boix et al., 2018). Dual stance is the duration of ground contact for both hind paws simultaneously in seconds. The catwalk analyzes initial and terminal dual stances. Another parameter that could reflect FoG is the duty cycle, which stands as a percentage of the step cycle. Stand is the duration of contact with the ground of the paw. The duration of “no contact” is the swing. The sum of stand + swing is the step cycle.

FoG would increase the percentage of time that the paw remains in contact with the ground, that is, the duty cycle. Golexanolone reverses the increase in initial dual stance, the increase in duty cycle, and the decrease in swing in front paws in 6-OHDA rats. This suggests that golexanolone could improve the freezing of gait in patients with Parkinson's.

Concerning non-motor symptoms, golexanolone improves the key symptoms that contribute to impairing the quality of life of the patients: fatigue, anxiety, depression, and short-term memory.

Fatigue is one of the most common and disabling symptoms in patients with PD, which has a high prevalence and has a significant impact on their quality of life (Beiske and Svensson, 2010; Kostić et al., 2016). Approximately 35% of PD patients show depression and anxiety, the most common psychiatric symptoms of PD (Zhang et al., 2023).

Heitmann et al. (2022) propose that neuroinflammation plays a key role in fatigue and depression in multiple sclerosis. Similar mechanisms may contribute to inducing fatigue and depression in PD. The reduction of glial activation and neuroinflammation by golexanolone may contribute to the improvement of fatigue and depression.

Cognitive impairment in patients with PD is also one of the most common disabling non-motor manifestations during the course of the disease. Neuroinflammation has also been proposed as a target to improve cognitive impairment in PD (Degirmenci et al., 2023), and golexanolone may improve short-term memory by reducing glial activation and neuroinflammation.

Concerning the mechanisms by which golexanolone improves motor and non-motor symptoms in the 6-OHDA rats, three main contributors would be: (1) reduction of microglia and astrocyte activation; (2) reduction of the loss of TH in the striatum; and (3) prevention of the increase in the content of α-synuclein. Golexanolone reduced glial activation and TH loss transiently; the beneficial effects were remarkable at 5 weeks after surgery but were lost at 10 weeks after surgery as brain damage progressed further. However, this was enough to improve motor and non-motor symptoms even at 8 weeks after surgery and potentially for longer periods. The prevention of the increase in the content of α-synuclein by golexanolone appears to be long-lasting and may also contribute to the sustained improvement of motor and non-motor symptoms.

An additional possible mechanism involved in the beneficial effects of golexanolone would be the reduction of GABA_A_ receptor activation, which would reduce the inhibitory tone acting on surviving dopamine neurons, thereby increasing dopaminergic neuron activity, which would improve functional outcomes. This may explain the improvement in golexanolone behavior at 10 weeks post-lesion.

The activation of microglia and astrocytes plays a key role in the pathology of PD and other α-synucleinopathies (Kam et al., 2020; Stefanova, 2022; Chen et al., 2023). Golexanolone reduces microglia and astrocyte activation at 5 weeks after surgery. We have shown that golexanolone also reverses microglia and astrocyte activation in the rats with hyperammonemia and hepatic encephalopathy, and this is associated with reduced neuroinflammation and improvement of cognitive and motor function (Mincheva et al., 2022; Arenas et al., 2023). A similar reduction of glial activation is induced by bicuculline, an antagonist of GABA_A_ receptors (Malaguarnera et al., 2019, 2021), indicating that the reduction by golexanolone of glial activation is likely mediated by the reduction of activation of GABA_A_ receptors.

Golexanolone also significantly reduces the loss of TH in the striatum at 5 weeks after surgery. The mechanism involved would be similar to that reported by Heo et al. (2020). As detailed in the introduction, they showed that GABA levels are increased in activated astrocytes of the substantia nigra in the rat models of PD, leading to enhanced activation of GABA_A_ receptors in neurons, which, in turn, reduces the expression of TH in “dormant” but alive neurons. Reducing GABA levels with MAO-B inhibitors or blocking GABA_A_ receptors reverses the loss of TH and improves motor symptoms in these rats (Heo et al., 2020). Golexanolone would prevent TH loss by similar mechanisms, reducing the activation of GABA_A_ receptors and thus increasing TH expression. This is associated with improvements in motor and non-motor symptoms in 6-OHDA rats. Golexanolone does not prevent the loss of TH at 10 weeks after surgery, probably due to the progression of neuronal damage. However, the transient prevention of TH loss by golexanolona is enough to afford a long-lasting improvement in behavioral deficits.

Golexanolone also prevents the increase of α-synuclein at 10 weeks after surgery. Thus, α-synuclein may play an important role in the non-motor symptoms of PD (Jellinger, 2011). The prevention of the increase of α-synuclein could contribute to the improvement by golexanolone of motor and non-motor symptoms in 6-OHDA rats.

The mechanism by which golexanolone prevents the increase in α-synuclein may also be mediated by the reduction of GABA_A_ receptor activation (Ross et al., 2020). Microglia activation facilitates the transmission of α-synuclein in PD (Zheng and Zhang, 2021). The reduction of microglia activation by golexanolone could also contribute to preventing the increase of α-synuclein in 6-OHDA rats.

In summary, we show that golexanolone improves some motor symptoms, motor incoordination, and some aspects of locomotor gait; it seems to improve the freezing of gait and improves key non-motor symptoms such as fatigue, depression, anxiety, and short-term memory, which strongly impair the quality of life of the patients. Moreover, golexanolone treatment does not induce dyskinesia in the 6-OHDA rat model, a behavior reported for L-DOPA that seriously impairs quality of life. Altogether, the improvement of all these symptoms by golexanolone would significantly improve the quality of life of patients with PD. The mechanisms by which golexanolone exerts these beneficial effects include a transient but very important reduction of microglia, astrocyte activation of tyrosine hydroxylase loss, and the sustained prevention of α-synuclein accumulation.

Golexanolone has already been shown to be safe and may be used in patients (Montagnese et al., 2021). This preclinical study shows that golexanolone is a promising therapeutic drug to improve fatigue, anxiety, depression, and cognitive and motor function in patients with PD. The findings underscore the necessity of conducting additional studies with patients to confirm the therapeutic utility of golexanolone.

## Data availability statement

The raw data supporting the conclusions of this article will be made available by the authors, without undue reservation.

## Ethics statement

The animal study was approved by the Comité de Etica y Experimentación Animal (CEEA) of Principe Felipe Research Centre and Conselleria de Agricultura, Generalitat Valenciana. The study was conducted in accordance with the local legislation and institutional requirements.

## Author contributions

PI-A: Writing – original draft, Visualization, Validation, Investigation, Formal analysis. YA: Writing – original draft, Investigation, Formal analysis. MM-G: Writing – original draft, Methodology, Investigation. LV: Writing – original draft, Investigation. GM: Writing – original draft, Investigation, Formal analysis. MD: Writing – review & editing, Visualization, Validation, Funding acquisition, Conceptualization. TB: Writing – review & editing, Visualization, Validation, Funding acquisition, Conceptualization. NB: Writing – review & editing, Visualization, Conceptualization. ML: Writing – review & editing, Visualization, Validation, Supervision, Formal analysis. VF: Writing – review & editing, Writing – original draft, Visualization, Supervision, Project administration, Funding acquisition, Conceptualization.

## References

[B1] Agusti A. Hernández-Rabaza V. Balzano T. Taoro-Gonzalez L. Ibañez-Grau A. Cabrera-Pastor A.. (2017). Sildenafil reduces neuroinflammation in cerebellum, restores GABAergic tone, and improves motor in-coordination in rats with hepatic encephalopathy. CNS Neurosci. Ther. 23, 386–394. 10.1111/cns.12688 28296282 PMC6492705

[B2] Arenas Y. M. Izquierdo-Altarejos P. Martinez-García M. Giménez-Garzó C. Mincheva G. Doverskog M.. (2023). Golexanolone improves fatigue, motor incoordination and gait and memory in rats with bile duct ligation. Liver Int. 44, 433–445. 10.1111/liv.15782 38010893

[B3] Balestrino R. Schapira A. H. V. (2020). Parkinson disease. Eur. J. Neurol. 27, 27–42. 10.1111/ene.14108 31631455

[B4] Balzano T. Esteban-García N. Blesa J. (2023). Neuroinflammation, immune response and α-synuclein pathology: how animal models are helping us to connect dots. Expert. Opin. Drug Discov. 18, 13–23. 10.1080/17460441.2023.2160440 36538833

[B5] Beiske A. G. Svensson E. (2010). Fatigue in Parkinson's disease: a short update. Acta Neurol. Scand. Suppl. 190, 78–81. 10.1111/j.1600-0404.2010.01381.x 20586741

[B6] Bengtsson S. K. S. Sjöstedt J. Malinina E. Das D.overskog, M. Johansson M. Haage D.. (2023). Extra-Synaptic GABAA receptor potentiation and neurosteroid-induced learning deficits are inhibited by GR3027, a GABAA modulating steroid antagonist. Biomolecules 13:1496. 10.3390/biom13101496 37892178 PMC10604444

[B7] Boix J. von Hieber D. Connor B. (2018). Gait analysis for early detection of motor symptoms in the 6-OHDA rat model of Parkinson's disease. Front. Behav. Neurosci. 12:39. 10.3389/fnbeh.2018.00039 29559901 PMC5845681

[B8] Butterworth R. F. Lalonde R. Power C. Baker G. B. Gamrani H. Ahboucha S. (2009). Dehydroepiandrosterone sulphate improves cholestasis-associated fatigue in bile duct ligated rats. Neurogastroenterol. Motil. 21, 1319–1325. 10.1111/j.1365-2982.2009.01356.x 19594690

[B9] Carvalho M. M. Campos F. L. Coimbra B. Pêgo J. M. Rodrigues C. Lima R.. (2013). Behavioral characterization of the 6-hydroxidopamine model of Parkinson's disease and pharmacological rescuing of non-motor deficits. Mol. Neurodegener. 8:14. 10.1186/1750-1326-8-14 23621954 PMC3653696

[B10] Cenci M. A. Lundblad M. (2007). Ratings of L-DOPA-induced dyskinesia in the unilateral 6-OHDA lesion model of Parkinson's disease in rats and mice. Curr. Protoc. Neurosci. 9, 4–25. 10.1002/0471142301.ns0925s41 18428668

[B11] Chen K. Wang H. Ilyas I. Mahmood A. Hou L. (2023). Microglia and astrocytes dysfunction and key neuroinflammation-based biomarkers in Parkinson's disease. Brain Sci. 13:634. 10.3390/brainsci13040634 37190599 PMC10136556

[B12] Cui H. Elford J. D. Alitalo O. Perez-Pardo P. Tampio J. Huttunen K. M.. (2023). Nigrostriatal 6-hydroxydopamine lesions increase alpha-synuclein levels and permeability in rat colon. Neurobiol. Aging. 129, 62–71. 10.1016/j.neurobiolaging.2023.05.007 37271045

[B13] Degirmenci Y. Angelopoulou E. Georgakopoulou V. E. Bougea A. (2023). Cognitive impairment in Parkinson's disease: an updated overview focusing on emerging pharmaceutical treatment approaches. Medicina 59:1756. 10.3390/medicina59101756 37893474 PMC10608778

[B14] Dickson D. W. (2012). Parkinson's disease and parkinsonism: neuropathology. Cold Spring Harb. Perspect. Med. 2:a009258. 10.1101/cshperspect.a009258 22908195 PMC3405828

[B15] Dorsey E. R. Sherer T. Okun M. S. Bloem B. R. (2018). The emerging evidence of the Parkinson pandemic. J. Parkinson's Dis. 8, S3–S8. 10.3233/JPD-181474 30584159 PMC6311367

[B16] Felipo V. Miñana M. D. Azorín I. Grisolía S. (1988). Induction of rat brain tubulin following ammonium ingestion. J. Neurochem. 51, 1041–1045. 10.1111/j.1471-4159.1988.tb03065.x 3418342

[B17] Gonzalez-Perez O. Gonzalez-Castañeda R. E. Huerta M. Luquin S. Gomez-Pinedo U. Sanchez-Almaraz E.. (2002). Beneficial effects of alpha-lipoic acid plus vitamin E on neurological deficit, reactive gliosis and neuronal remodeling in the penumbra of the ischemic rat brain. Neurosci. Lett. 321, 100–104. 10.1016/S0304-3940(02)00056-3 11872266

[B18] Heitmann H. Andlauer T. F. M. Korn T. Mühlau M. Henningsen P. Hemmer B.. (2022). Fatigue, depression, and pain in multiple sclerosis: how neuroinflammation translates into dysfunctional reward processing and anhedonic symptoms. Mult. Scler. 28, 1020–1027. 10.1177/1352458520972279 33179588 PMC9131410

[B19] Heo J. Y. Nam M. H. Yoon H. H. Kim J. Hwang Y. J. Won W.. (2020). Aberrant tonic inhibition of dopaminergic neuronal activity causes motor symptoms in animal models of Parkinson's disease. Curr. Biol. 30, 276–291.e9. 10.1016/j.cub.2019.11.079 31928877

[B20] Jellinger K. A. (2011). Synuclein deposition and non-motor symptoms in Parkinson disease. J. Neurol. Sci. 310, 107–111. 10.1016/j.jns.2011.04.012 21570091

[B21] Johansson M. Agusti A. Llansola M. Montoliu C. Strömberg J. Malinina E.. (2015). GR3027 antagonizes GABAA receptor-potentiating neurosteroids and restores spatial learning and motor coordination in rats with chronic hyperammonemia and hepatic encephalopathy. Am. J. Physiol. Gastrointest. Liver Physiol. 309, G400–G409. 10.1152/ajpgi.00073.2015 26138462 PMC4556948

[B22] Johansson M. Månsson M. Lins L.-E. Scharschmidt B. Doverskog M. Bäckström T. (2018). GR3027 reversal of neurosteroid-induced, GABA-A receptor-mediated inhibition of human brain function: an allopregnanolone challenge study. Psychopharmacology 235, 1533–1543. 10.1007/s00213-018-4864-1 29492615 PMC5919995

[B23] Johansson M. Strömberg J. Ragagnin G. Doverskog M. Bäckström T. (2016). GABAA receptor modulating steroid antagonists (GAMSA) are functional in vivo. J. Steroid Biochem. Mol. Biol. 160, 98–105. 10.1016/j.jsbmb.2015.10.019 26523675

[B24] Kam T. I. Hinkle J. T. Dawson T. M. Dawson V. L. (2020). Microglia and astrocyte dysfunction in parkinson's disease. Neurobiol. Dis. 144:105028. 10.1016/j.nbd.2020.105028 32736085 PMC7484088

[B25] Kostić V. S. Tomić A. Ječmenica-Lukić M. (2016). The pathophysiology of fatigue in Parkinson's disease and its pragmatic management. Mov. Disord. Clin. Pract. 3, 323–330 10.1002/mdc3.12343 30363584 PMC6178705

[B26] Leão A. H. F. F. Meurer Y. S. R. Freitas T. A. Medeiros A. M. Abílio V. C. Izídio G. S.. (2021). Changes in the mesocorticolimbic pathway after low dose reserpine-treatment in Wistar and Spontaneously Hypertensive Rats (SHR): Implications for cognitive deficits in a progressive animal model for Parkinson's disease. Behav. Brain Res. 410:113349. 10.1016/j.bbr.2021.113349 33971246

[B27] Lemos J. C. Friend D. M. Kaplan A. R. Shin J. H. Rubinstein M. Kravitz A. V.. (2016). Enhanced GABA transmission drives bradykinesia following loss of dopamine D2 receptor signaling. Neuron. 90:824–838. 10.1016/j.neuron.2016.04.040 27196975 PMC4882167

[B28] Lindgren H. S. Dunnett S. B. (2012). Cognitive dysfunction and depression in Parkinson's disease: what can be learned from rodent models? Eur. J. Neurosci. 35, 1894–1907. 10.1111/j.1460-9568.2012.08162.x 22708601

[B29] Lundblad M. Andersson M. Winkler C. Kirik D. Wierup N. Cenci M. A. (2002). Pharmacological validation of behavioural measures of akinesia and dyskinesia in a rat model of Parkinson's disease. Eur. J. Neurosci. 15, 120–132. 10.1046/j.0953-816x.2001.01843.x 11860512

[B30] Malaguarnera M. Balzano T. Castro M. C. Llansola M. Felipo V. (2021). The dual role of the GABAA receptor in peripheral inflammation and neuroinflammation: a study in hyperammonemic rats. Int. J. Mol. Sci. 22:6772. 10.3390/ijms22136772 34202516 PMC8268725

[B31] Malaguarnera M. Llansola M. Balzano T. Gómez-Giménez B. Antúnez-Muñoz C. Martínez-Alarcón N.. (2019). Bicuculline reduces neuroinflammation in hippocampus and improves spatial learning and anxiety in hyperammonemic rats. role of glutamate receptors. Front. Pharmacol. 10:132. 10.3389/fphar.2019.00132 30858801 PMC6397886

[B32] Mincheva G. Gimenez-Garzo C. Izquierdo-Altarejos P. Martinez-Garcia M. Doverskog M. Blackburn T. P.. (2022). Golexanolone, a GABAA receptor modulating steroid antagonist, restores motor coordination and cognitive function in hyperammonemic rats by dual effects on peripheral inflammation and neuroinflammation. CNS Neurosci. Ther. 28, 1861–1874. 10.1111/cns.13926 35880480 PMC9532914

[B33] Montagnese S. Lauridsen M. Vilstrup H. Zarantonello L. Lakner G. Fitilev S.. (2021). A pilot study of golexanolone, a new GABA-A receptor-modulating steroid antagonist, in patients with covert hepatic encephalopathy. J. Hepatol. 75, 98–107. 10.1016/j.jhep.2021.03.012 33894327

[B34] Muñoz M. D. de la Fuente N. Sánchez-Capelo A. (2020). TGF-β/Smad3 signalling modulates GABA neurotransmission: implications in Parkinson's disease. Int. J. Mol. Sci. 21:590. 10.3390/ijms21020590 31963327 PMC7013528

[B35] Rodrigo R. Cauli O. Gomez-Pinedo U. Agusti A. Hernandez-Rabaza V. Garcia-Verdugo J. M.. (2010). Hyperammonemia induces neuroinflammation that contributes to cognitive impairment in rats with hepatic encephalopathy. Gastroenterology 139, 675–684. 10.1053/j.gastro.2010.03.040 20303348

[B36] Ross A. Xing V. Wang T. T. Bureau S. C. Link G. A. Fortin T.. (2020). Alleviating toxic α-Synuclein accumulation by membrane depolarization: evidence from an in vitro model of Parkinson's disease. Mol. Brain 13:108. 10.1186/s13041-020-00648-8 32736645 PMC7395353

[B37] Schneider J. S. Marshall C. A. Keibel L. Snyder N. W. Hill M. P. Brotchie J. M.. (2021). A novel dopamine D3R agonist SK609 with norepinephrine transporter inhibition promotes improvement in cognitive task performance in rodent and non-human primate models of Parkinson's disease. Exp. Neurol. 335:113514. 10.1016/j.expneurol.2020.113514 33141071 PMC7750278

[B38] Stefanova N. (2022). Microglia in Parkinson's disease. J. Parkinsons. Dis. 12, S105–S112. 10.3233/JPD-223237 35754289 PMC9535597

[B39] Tadaiesky M. T. Dombrowski P. A. Figueiredo C. P. Cargnin-Ferreira E. Da Cunha C. Takahashi R. N. (2008). Emotional, cognitive and neurochemical alterations in a premotor stage model of Parkinson's disease. Neuroscience 156, 830–840. 10.1016/j.neuroscience.2008.08.035 18817851

[B40] Tian Q. Tang H. L. Tang Y. Y. Zhang P. Kang X. Zou W.. (2022). Hydrogen sulfide attenuates the cognitive dysfunction in Parkinson's disease rats via promoting hippocampal microglia M2 polarization by enhancement of hippocampal warburg effect. Oxid. Med. Cell. Longev. 2022:2792348. 10.1155/2022/2792348 35028004 PMC8752224

[B41] Torres E. M. Dunnett S. B. (2011). “6-OHDA lesion models of Parkinson's disease in the Rat,” in Animal Models of Movement Disorders. Neuromethods, eds. E. Lane, S. Dunnett (Totowa, NJ: Humana Press). 10.1007/978-1-61779-298-4_13

[B42] Tran T. N. Vo T. N. N. Frei K. Truong D. D. (2018). Levodopa-induced dyskinesia: clinical features, incidence, and risk factors. J. Neural. Transm. 125, 1109–1117. 10.1007/s00702-018-1900-6 29971495

[B43] Ujvári B. Pytel B. Márton Z. Bognár M. Kovács L. Á. Farkas J.. (2022). Neurodegeneration in the centrally-projecting Edinger-Westphal nucleus contributes to the non-motor symptoms of Parkinson's disease in the rat. J. Neuroinflam. 19:31. 10.1186/s12974-022-02399-w 35109869 PMC8809039

[B44] Ullah F. Asgarov R. Venigalla M. Liang H. Niedermayer G. Münch G.. (2020). Effects of a solid lipid curcumin particle formulation on chronic activation of microglia and astroglia in the GFAP-IL6 mouse model. Sci. Rep. 10:2365. 10.1038/s41598-020-58838-2 32047191 PMC7012877

[B45] Xiao H. Li M. Cai J. Li N. Zhou M. Wen P.. (2017). Selective cholinergic depletion of pedunculopontine tegmental nucleus aggravates freezing of gait in parkinsonian rats. Neurosci. Lett. 659, 92–98. 10.1016/j.neulet.2017.08.016 28803956

[B46] Zhang T. Yang R. Pan J. Huang S. (2023). Parkinson's disease related depression and anxiety: a 22-year bibliometric analysis (2000-2022). Neuropsychiatr. Dis. Treat. 19, 1477–1489. 10.2147/NDT.S403002 37404573 PMC10317541

[B47] Zheng T. Zhang Z. (2021). Activated microglia facilitate the transmission of α-synuclein in Parkinson's disease. Neurochem. Int. 148:105094. 10.1016/j.neuint.2021.105094 34097990

[B48] Zhou Z. D. Saw W. T. Ho P. G. H. Zhang Z. W. Zeng L. Chang Y. Y.. (2022). The role of tyrosine hydroxylase-dopamine pathway in Parkinson's disease pathogenesis. Cell. Mol. Life Sci. 79:599. 10.1007/s00018-022-04574-x 36409355 PMC9678997

[B49] Zörner B. Filli L. Starkey M. L. Gonzenbach R. Kasper H. Röthlisberger M.. (2010). Profiling locomotor recovery: comprehensive quantification of impairments after CNS damage in rodents. Nat. Methods. 7, 701–708. 10.1038/nmeth.1484 20836253

