# Supplementary figures and images for: Golexanolone reduces glial activation in the striatum and improves non-motor and some motor alterations in a rat model of Parkinson's disease
Source: Front Aging Neurosci. 2024 Jun 21;16:1417938. doi: 10.3389/fnagi.2024.1417938 (PMC11224447; doi:10.3389/fnagi.2024.1417938)

Figure 5E

Molecular weight  
marker (KDa)

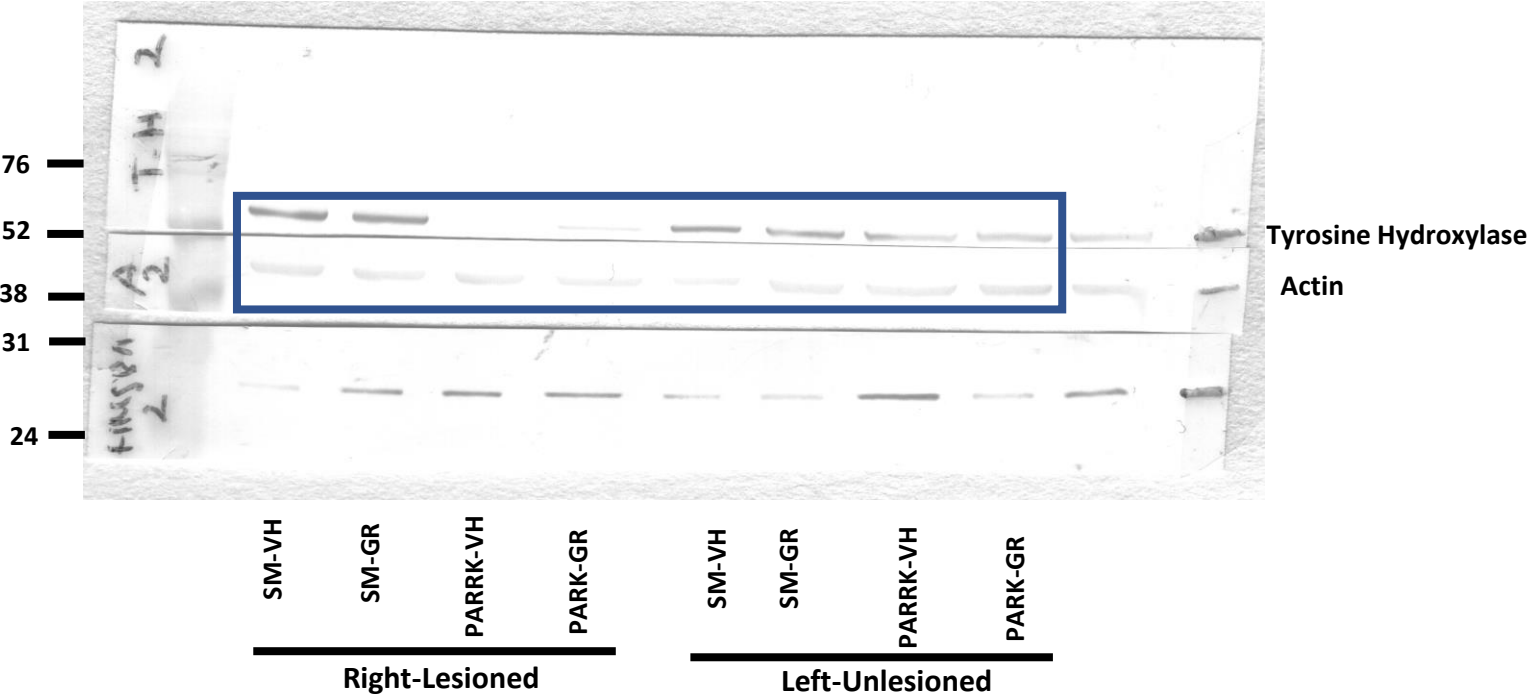

### Figure 6C

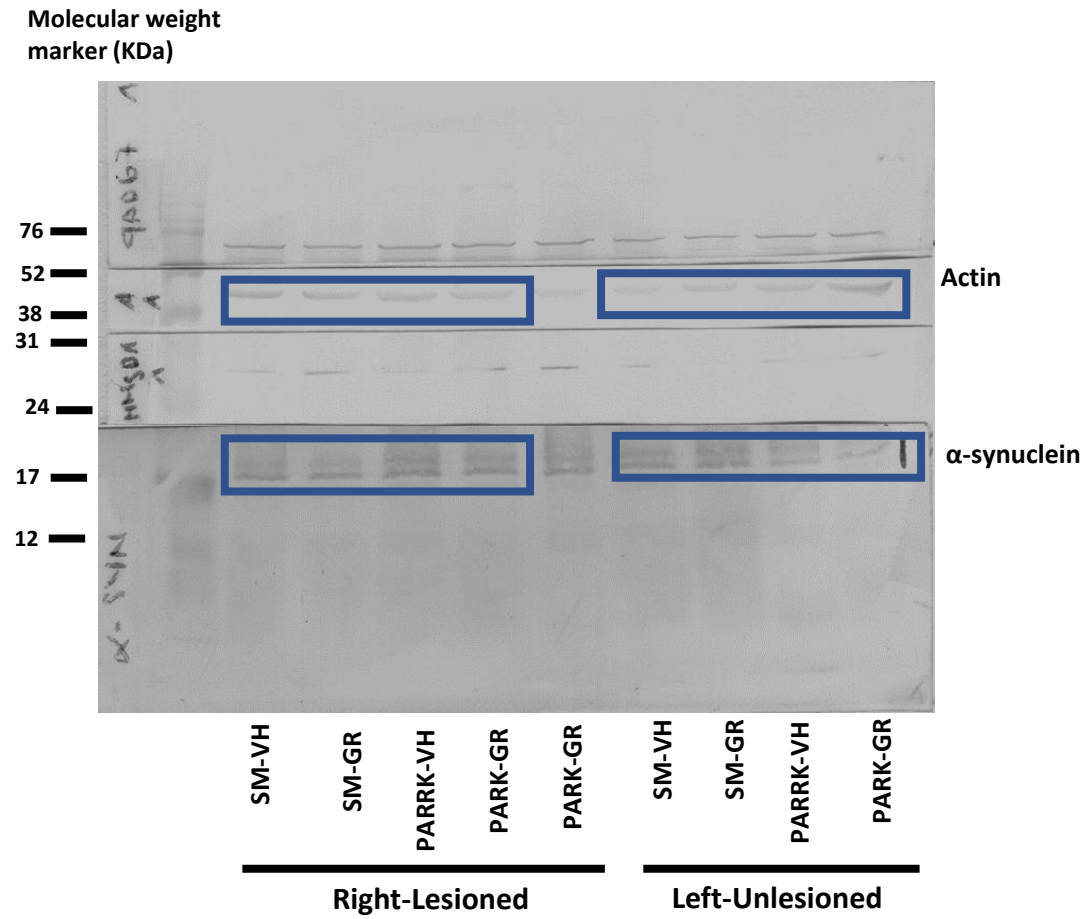

Supplement: Supplementary file 1 [file Data_Sheet_1.PDF]
